# Genome-wide analysis of MAPKKKs shows expansion and evolution of a new MEKK class involved in solanaceous species sexual reproduction

**DOI:** 10.1186/s12864-015-2228-3

**Published:** 2015-12-09

**Authors:** Caroline Daigle, Daniel P. Matton

**Affiliations:** Institut de Recherche en Biologie Végétale, Département de Sciences Biologiques, Université de Montréal, 4101 rue Sherbrooke est, Montréal, H1X 2B2 QC Canada

**Keywords:** Mitogen-Activated Protein Kinase Kinase Kinases (MAPKKKs), MEKKs, Fertilization-Related Kinases (FRKs), *Solanaceae*, Sexual reproduction, Evolution

## Abstract

**Background:**

Members of the plant MAP Kinases superfamily have been mostly studied in *Arabidopsis thaliana* and little is known in most other species. In *Solanum chacoense*, a wild species close to the common potato, it had been reported that members of a specific group in the MEKK subfamily, namely ScFRK1 and ScFRK2, are involved in male and female reproductive development. Apart from these two kinases, almost nothing is known about the roles of this peculiar family.

**Methods:**

MEKKs were identified using BLAST and hidden Markov model (HMM) to build profiles using the 21 MEKKs from *A. thaliana*. Following protein sequence alignments, the neighbor-joining method was used to reconstruct phylogenetic trees of the MEKK subfamily. Kinase subdomains sequence logos were generated with WebLogo in order to pinpoint FRK distinct motifs. Codon alignments of the FRKs kinase subdomains and maximum-likelihood phylogenetic trees were used in the codon substitution models of the codeml program in the PAML package to detect selective pressure between FRK groups.

**Results:**

With the recent progress in Next-Generation Sequencing technologies, the genomes and transcriptomes of numerous plant species have been recently sequenced, giving access to a vast amount of data. With the aim of finding all members of the MEKK subfamily members in plants, we screened the genomes of 15 species from different clades of the plant kingdom. Interestingly, the whole MEKK subfamily has significantly expanded throughout evolution, especially in solanaceous species. This holds true for members of the FRK class, which have also strongly expanded and diverged.

**Conclusions:**

Expansion and rapid evolution of the FRK class members in solanaceous species support the hypothesis that they have acquired new roles, mainly in male and female reproductive development.

**Electronic supplementary material:**

The online version of this article (doi:10.1186/s12864-015-2228-3) contains supplementary material, which is available to authorized users.

## Background

Protein phosphorylation is the most widespread post-translational modification in eukaryotic cells [1]. Its pivotal role is exemplified by the vast array of cellular processes that it regulates and the preponderance of protein kinases (PKs) in the cell proteome, even more for plants, where PKs represent around 4 % of the proteome, a two-fold increase compared to other eukaryotes [2]. In plants, mitogen-activated protein kinases (MAPKs) are one of the largest PKs family, second only to the receptor kinases superfamily. MAPKs form signaling cascades that can quickly transfer information from upstream sensors to downstream effectors, allowing cells to mount an appropriate response. They are minimally composed of three distinct modules that are widely conserved among eukaryotes. Once activated, the first member of the signaling cascade, the mitogen-activated protein kinase kinase kinase (MAPKKK, MKKK or MEKK) phosphorylates and activates the second member, a mitogen-activated protein kinase kinase (MAPKK or MKK) on a [S/T] - X_3–5_ - [S/T] motif (X = 5 in plants; X = 3 in animal and yeast) [3, 4]. The activated MKK can then phosphorylate the third player, the mitogen-activated protein kinase (MAPK or MPK), on a T - [D/E] - Y motif located in the MAPK activation loop. Once activated, the MAPK can phosphorylate a variety of downstream target proteins including transcription factors, other kinases and cytoskeleton-associated proteins [5]. Activation of the MAPKKK itself most often occurs through the intervention of cell surface receptors. Upon ligand binding, receptor-mediated activation of the MAPKKK can proceed through direct or indirect phosphorylation (through a mitogen-activated protein kinase kinase kinase kinase (MAPKKKK), or by a physically induced conformational changes [6]. Although MAPK pathways have been intensively studied in yeast, drosophila and mammalian cells, functional characterization of canonical MAPK pathways in plants is still in its infancy. Most of our knowledge about plant MAPKs cascades comes from the model species *Arabidopsis thaliana,* the first plant to have its genome sequenced [7]. Although *A. thaliana* contains 10 MAPKKKKs, around 80 MAPKKKs (divided into three subfamilies, the MEKKs, the RAFs and the ZIKs) [8], 10 MAPKKs and 20 MAPKs [3, 9], only few complete cascades have been deciphered. These include the MEKK1 - MKK4/5 – MPK3/6 cascade that positively regulates plant innate immunity [10] and also acts in phytoalexin biosynthesis [11]. MEKK1 with different partners, MKK1/2 – MPK4, also acts in an independent signaling cascade involved in stress signaling such as oxidative, salt and cold stresses [12, 13] as well as in defense responses against pathogens [14] and innate immunity [15]. Very recently, another complete cascade involving MKKK17/18-MKK3-MPK1/2/7/14 was also shown to be involved in stress signaling under the control of the phytohormone abscisic acid (ABA) [16]. Signaling cascades involved in plant development have also been uncovered, like the YODA – MKK4/5 – MPK3/6 cascade involved in both stomatal and embryonic development [17–20]. The YODA signaling pathway was the first well-described MAPK signaling pathway acting in plant reproductive development. In the *yda* mutant, it was shown that the zygote could not divide properly, leading to inappropriate cell divisions of the suspensor and, eventually, to the development of an embryo without the root primordium [20].

No complete MAPK signaling cascade has yet been reported in plant species other than *A. thaliana*, with the exception of the tobacco NACK-PQR cascade involved in sporophytic cytokinesis [21–23], but with the recent advent of Next-Generation Sequencing (NGS) technologies, genomes and transcriptomes from rice (*Oryza sativa*) [24], maize (*Zea mays*) [25], poplar (*Populus trichocarpa*) [26], potato (*Solanum tuberosum*) [27], tomato (*Solanum lycopersicum*) [28] and many other species are now available. This enables kinome comparison between genomes in order to trace their evolutionary histories. Comparative phylogenetic studies of MAPKs families revealed that there were only few differences in sequences and numbers of family members among the studied species [29–35]. Similarly to *Arabidopsis*, 75 MAPKKKs, 8 MKKs and 17 MAPKs were identified in rice [33, 35]. Sequence identity between MPKs from these two and others species is very high, suggesting a high degree of conservation between the MPK family [29, 30, 32, 35–38]. High amino acid sequence identity was also observed within orthologous MKKs from numerous species [29, 33, 35, 37]. The MAPKKKs however, showed high sequence diversity, mainly outside the kinase domain. Compared to the MKKs and MPKs, most MAPKKKs comprise long N or C-terminal extensions harboring regions that play specific roles including kinase activity control, selection of their cognate MKKs or act as scaffolds for the recruitment of other proteins in the signaling cascade [39]. Among the MAPKKKs, the MEKK subfamily is the only one known to be involved in canonical MKKK-MKK-MPK cascades, and one group departs from the others having no lengthy N or C-terminal extensions. In *A. thaliana*, this group comprises three closely related MEKKs, the AtMAPKKK 19, 20 and 21 (At5g67080, At3g50310 and At4g36950, respectively). They exhibit from 56 to 75 % pairwise amino acid sequence identity (70–86 % similarity), and are much smaller than all other MEKKs ranging from 336 to 344 amino acids (MW ~37 kDa). Although little information about these proteins is available, evidences show that AtMAPKKK20 interacts with numerous calmodulins and calmodulin-like proteins [40] and is involved in osmotic stress responses [41]. As a target of the DUO1 transcription factor involved in sperm cell differentiation [42], *AtMAPKKK20* is most probably also involved in pollen development. It was also recently shown that the orthologs of *AtMAPKKK19* and *20* in *Brassica napus* are also regulated by stress responses [43]. Three putative orthologs of these MAPKKKs had been identified in *Solanum chacoense,* a wild diploid potato species close to *Solanum tuberosum*: ScFRK1, ScFRK2 and ScFRK3 (for *Solanum chacoense* Fertilization-Related Kinase) [44, 45]. All three are involved in plant sexual reproduction, affecting both male and female floral organs. In plants overexpressing *ScFRK2*, a homeotic transformation of the integument into a carpelloid structure leads to severely reduced seed set [46]. The female gametophyte, the embryo sac, was also affected (Daigle, C., Mazin, B., Matton, D. P. unpublished results). Pollen viability is also severely reduced in these transgenic lines [47]. RNAi and cosuppression transgenic lines for *ScFRK1* [48] and *ScFRK3* (Daigle et al., unpublished results) also showed severe defects in female gametophytes due to abnormal progression through megagametogenesis, leading to embryo sac collapse. Pollen viability is also severely reduced in those mutants. Except for the work done in *S. chacoense, A. thaliana* and *Brassica napus*, nothing else is known about these MAPKKKs in others species.

With the aim of building full MAPK cascades acting in plant reproduction, a deep transcriptomic analysis was performed on pollen and ovule samples in *S. chacoense*. Surprisingly, analysis of the MEKK subfamily showed a two-fold increase from the FRK group in *S. chacoense* compared to *A. thaliana*, with six different FRKs (*ScFRK1-6*) retrieved. Considering the highly specialized roles of the ScFRKs in plant reproductive development, this prompted us to analyze when this expansion arose during plant evolution by revisiting the MEKK classification in other species. In total, 15 genomes were analyzed, identifying the solanaceous species as harboring the largest MEKK subfamily. Furthermore, inside the plant MEKK subfamily, the proportion of FRKs among solanaceous species was two to three times higher than in other species. This expansion unique to solanaceous species suggests the emergence of new specific roles, particularly in reproductive development. This study highlights the compendium of all MEKK subfamily members from various clades in the plant kingdom and uncovers an important family involved in reproductive development.

## Methods

### RNA sequencing and de novo assembly

*Solanum chacoense* Bitt. plants (genotype G4, S_12_S_14_ self-incompatibility alleles) were greenhouse-grown under long-day condition (16 h light/8 h dark). The 454 GS-FLX Titanium next generation sequencing (NGS) platform was used to perform RNA-seq (one full plate per sample producing between 1 and 1,5 million reads each) on wild-type ovules at anthesis, ovules two days before anthesis, and anthesis ovules from the *ScFRK1* embryo sac-less mutant [48], as well as from mature dry pollen and from in vitro grown pollen tubes. For ovule collection, ovaries were hand dissected to remove the pericarp (ovary wall) and ovules were snap frozen in liquid nitrogen until use. Pollen was collected from mature anthers using a manual or electric vibrator device, while pollen tubes were obtained from germinating pollen grains in BK liquid medium for 5 h and collected on a 0.45 μM cellulose filter through gentle vacuum filtration [49]. Total RNA was extracted using the TRIzol reagent (Life Technologies; Cat No 15596–026) as recommended by the manufacturer. cDNA libraries were constructed for each condition with the GS FLX Titanium Rapid Library Preparation Kit (Roche; Cat No 05608228001) after an mRNA enrichment step performed with Dynabeads Oligo(dT)_25_ (Life Technologies; Cat No 25–61002). Long reads obtained from the 454 platform at The Center for Applied Genomics (TCAG, Toronto, Canada) were *de novo* assembled into 2141 contigs and 51 162 isotigs leading to 26 838 isogroups using the Newbler software (Roche) [50]. The 53 303 sequences are available at http://www.ncbi.nlm.nih.gov/Traces/wgs/?val=GDZX01.

### *S. chacoense* MEKK subfamily analysis

The assembled transcriptomes of the four samples were merged together and the whole 454 database was screened to retrieve all members of the MEKK subfamily. Amino acids sequences from the kinase domains of the 21 MEKKs in *A. thaliana* were used to screen against the *S. chacoense* transcriptome. The BLAST algorithm (tblastn) was used to compare the *A. thaliana* sequences against the *S. chacoense* database. The matching transcripts (20 best hits for each *A. thaliana* MEKKs screened) were then compared with the NCBI database to ensure that they corresponded to true MEKKs. Pfam 27.0 (http://pfam.sanger.ac.uk/) and HMM (HMMER 3.1b1; hmmer.org) analyses were also used to ascertain their status as MEKKs. In total, 21 different MEKKs were found in the *S. chacoense* combined ovule and pollen transcriptome. Protein sequences of those 21 ScMEKKs can be found in Additional file 1: Table S1. Sequence alignments were made with ClustalW and all phylogenetic trees were performed with the Neighbor-Joining algorithm with a 1000 bootstrap replicates (only bootstrap value >50 % are mentioned) and rooted with the kinase domain sequence of the *A. thaliana* BRI1 receptor kinase (At4g39400) in Geneious 8.0 (www.geneious.com).

### MEKK subfamily analysis in other species

Genomes, transcriptomes, cDNAs, CDS or peptides databases were downloaded and used to retrieve MEKK sequences corresponding to the *Arabidopsis* MEKK subfamily, and further validated as done previously for the *S. chacoense* transcriptome. Data resources retrieved from the other 13 species tested are listed in Additional file 1: Table S2. Genes ID and locus tags of all the MEKKs found in the databases are listed in Additional file 1: Table S3.

### Expression analysis of the FRK class in *S. chacoense*

Total RNA was isolated with the TRIzol® Reagent from Life Technologies (Cat. No 15596–026). Reverse transcription from 2.5 μg of total RNA extracted from various tissues was performed using the Moloney Murine Leukemia Virus Reverse Transcriptase (M-MLV RT) from Invitrogen (Cat. No 28025). PCR amplification cycles were as follows: 25 cycles for the actin gene; 27 cycles for *ScFRK1* and *2;* 28 cycles for *ScFRK3, 4 and 5;* and 39 cycles for *ScFRK6.* To ascertain RT-PCR primers specificity for the *ScFRK1-6* genes, the forward primer was positioned in the C-terminal domain following the kinase domain where pairwise nucleotide sequence identity varied from 19 to 71 %. The reverse primer was designed using the highly divergent 3′ UTR region where pairwise nucleotide sequence identity varied from 29 to 54 %. Primers shared lesser than 60 % identity between the *ScFRK1-6* genes. PCR primers and amplicons size are listed in Additional file 1: Table S4.

### Expression analysis of the MEKK subfamily in *S. lycopersicum*, *S. tuberosum* and *P. trichocarpa*

Expression analysis data were taken from the Bio-Analytic Resources for Plant Biology (BAR, http://bar.utoronto.ca/welcome.htm). For *S. tuberosum*, gene names (PGSC0003DMTXXXXXXXXX) were retrieved from the Sol Genomics Network website (http://solgenomics.net/). For *S. lycopersicum*, the Solyc code (SolycXXgXXXXXX) was used. For poplar, microarray IDs corresponding to their orthologous FRK proteins were retrieved from the Plant Compliant Gene Expression Resources for Plants and Plant Pathogens (PLEXdb) website (http://www.plexdb.org/index.php).

### Kinase domains sequence logos

Sequence alignments of all AtMAPKKK1-12 orthologs were used to create a sequence logo using the WebLogo website (http://weblogo.berkeley.edu/logo.cgi) in order to compare the 12 kinase subdomains. The same procedure was applied to AtMAPKKK13-14 orthologs, the AtMAPKKK15-18 orthologs and all the FRK-like proteins, separately. Sequence logos were also used to compare each group (groups I to IV) of the FRK class.

### Test of variable dN/dS ratios among the FRK class

Codon alignments of the FRKs 12 subdomains of the kinase domain and its Maximum-likelihood phylogenetic tree were used in the codon substitution models of the codeml program in the PAML package [51] to detect if there is a difference in selective pressure between group I and the three others groups. A branch-specific codon model was set to allow the dN/dS ratio (ω) to vary for group I. A likelihood ratio test (LRT) was used to determine whether there is a statistically significant difference in ω ratio or not.

## Results

### A deep transcriptomic analysis of the MEKK subfamily in *Solanum chacoense* unveils a much larger FRK class than in *Arabidopsis thaliana*

**Fig. 1:**
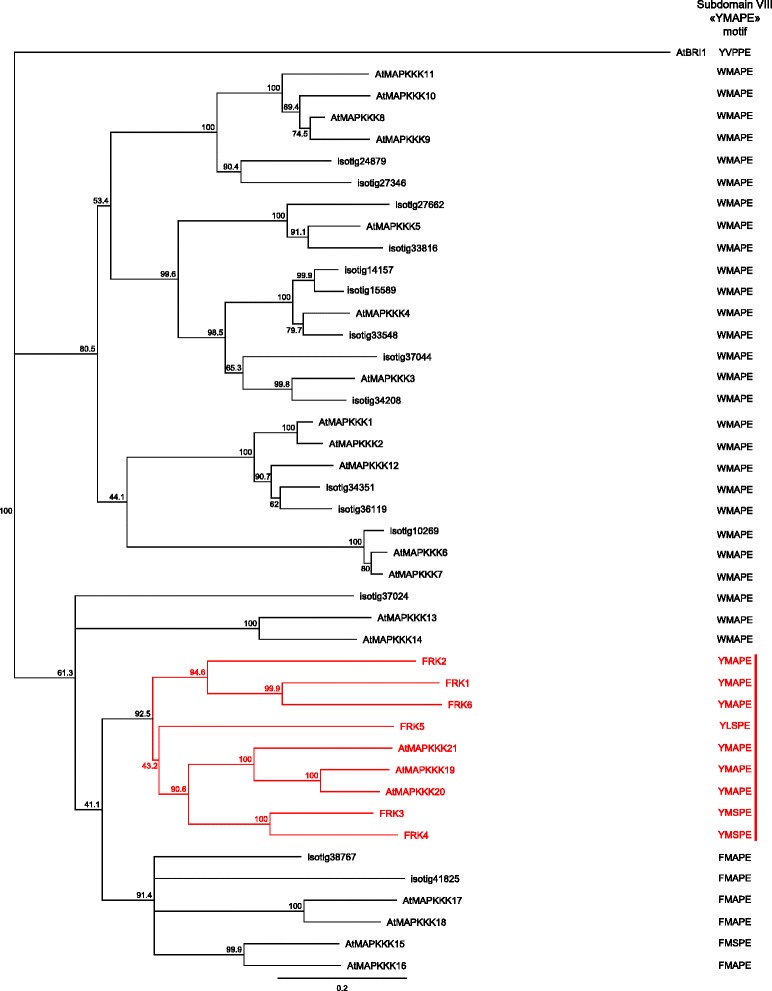
Phylogenetic analysis of the MEKK subfamily in *Arabidopsis thaliana* and *Solanum chacoense*. The phylogenetic tree at the left shows genetic relationships between the MEKKs from *A. thaliana* and the MEKKs found in the *S. chacoense* ovule and pollen tube transcriptomes. The YMAPE motif of each MAPKKK is shown on the right panel. The FRK class is shown in red. Gene IDs and locus tags can be found in Additional file 2: Table S2

The *A. thaliana* MEKK subfamily comprises 21 MAPKKKs with AtMAPKKK 19, 20 and 21 clustering with the *S. chacoense* FRKs, forming a highly supported clade. In order to find all *S. chacoense* MEKKs that could be involved in plant reproduction, we screened the *S. chacoense* ovule and pollen tube transcriptomes with each *A. thaliana* MEKKs. From 26,838 different transcripts, 21 different MEKKs were retrieved (Additional file 1: Table S1). Interestingly, apart from the already known FRKs, ScFRK1, 2 and 3 [44, 45], three new FRK members were found in the combined ovule/pollen transcriptome and named ScFRK4, 5 and 6. All six *S. chacoense* members grouped together in a phylogenetic tree with the AtMAPKKKs 19, 20 and 21. Apart from being the smallest MEKKs, they also share similar specific motifs like the “YMAPE” signature of subdomain VIII instead of the WMAPE or FMAPE signature present in the other MEKKs (Fig. 1). The family can be further divided in three groups: 1) one including ScFRK1, 2 and 6 with no *Arabidopsis* ortholog; 2) one including ScFRK3, 4 and AtMAPKKK19, 20 and 21; 3) and a third group represented by ScFRK5 alone.

### Comparison of the MEKK subfamily shows an expansion of the FRK class in Solanaceous species

**Fig. 2:**
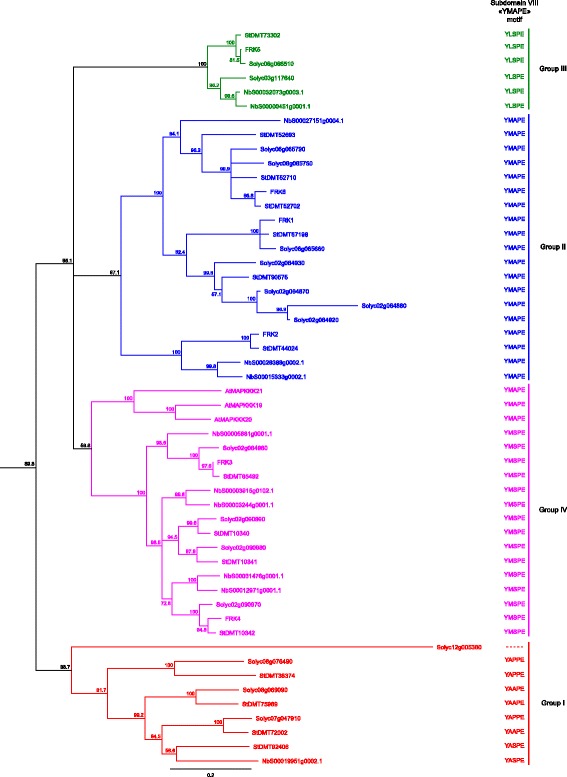
Phylogenetic analysis of the FRK class in solanaceous species. Phylogenetic relationships between members of the FRK class in *A. thaliana*, *S. chacoense*, *S. lycopersicum*, *S. tuberosum* and *N. benthamiana*. The four FRK subgroups are color coded in the tree. Gene IDs and locus tags can be found in Additional file 2: Table S2

To determine if the expansion of the FRK class is unique to the *Solanaceae*, the genomes of three recently sequenced solanaceous species (*Solanum lycopersicum*, *Solanum tuberosum* and *Nicotiana benthamiana*) were screened using the kinase domains of AtMEKKs. To simplify the phylogenetic analysis, only MEKKs that showed less than 95 % pairwise sequence identity within a species were considered. The others may result from recent duplication, be alleles or minor splice variants. As for *A. thaliana*, alignment of the 21 MEKKs kinase domains showed only a single pair of MEKKs (AtMAPKKK6 and AtMAPKKK7) with slightly more than 95 % identity (96,1 %), the others being lesser than 93 % identical. Interestingly, 39, 36 and 40 different MEKKs were retrieved from the *S. lycopersicum (Sl*), *S. tuberosum* (*St*) and *N. benthamiana* (*Nb*) genomes, respectively, which is almost twice than in *A. thaliana*. A phylogenetic analysis of the MEKK subfamily in these four solanaceous species is presented in Additional file 2: Figure S1 and a close-up of the FRK class is shown in Fig. 2. Of all the MEKKs found, 17 out of 39 SlMEKKs, 15 out of 36 StMEKKs and 11 out of 40 NbMEKKs were grouped in the FRK class. Compared to *Arabidopsis,* not only has the whole MEKK subfamily expanded in solanaceous species but the FRK class is also proportionally more preponderant. In *A. thaliana*, the FRK class represents only 14 % of the MEKKs (3/21) compared to 44 % (17/39) in *S. lycopersicum*, 42 % (15/36) in *S. tuberosum*, and 28 % (11/40) in *N. benthamiana*. The fact that the FRK class has expanded twice or thrice in solanaceous species compared to *A. thaliana* suggests the evolution of new and specific roles in these species.

Although the FRK class represents a highly supported clade, its members can be further clustered into four groups (Fig. 2). Group I (in red) comprised four MEKKs from *S. lycopersicum*, four from *S. tuberosum*, one from *N. benthamiana* but none from the *S. chacoense* ovule and pollen tube transcriptome. Group II (in blue) comprised seven MEKKs from *S. lycopersicum*, six from *S. tuberosum*, three from *N. benthamiana* and three from *S. chacoense* (ScFRK1, ScFRK2, ScFRK6). Group III (in green) comprised two MEKKs from *S. lycopersicum*, one from *S. tuberosum*, two from *N. benthamiana* and one from *S. chacoense* (ScFRK5). Group IV (magenta) comprised four MEKKs from *S. lycopersicum*, four from *S. tuberosum*, five from *N. benthamiana,* two from *S. chacoense* (ScFRK3 and ScFRK4), and the *Arabidopsis* MAPKKK19, 20, and 21. As for the AtMAPKKK19, 20 and 21, the whole FRK class shared the tyrosine (Y) in the YMAPE motif of subdomain VIII, with the exception of the *S. lycopersicum* Solyc12g005360 that completely lacked the motif, most probably leading to an inactive protein kinase. With its YAAPE, YAPPE, and YASPE motifs, group I displayed the most diversified motifs. Groups II and III encompassed MEKKs that harbor the *stricto sensu* YMAPE or the YLSPE motif, respectively, while group IV kinases had either the YMSPE or YMAPE motifs. The tyrosine in that motif is clearly a distinctive feature of the FRK class, not being found in any other MEKKs in solanaceous species or in *A. thaliana*, except for a group of four MEKKs in *N. benthamiana* that shares the YMAPE motif with the FRK class (see Additional file 2: Figure S1, group in burgundy). These four MEKKs are more or less related to AtMAPKKK15, 16, 17 and 18 that are the closest relatives to the AtMAPKKK19, 20 and 21.

### Uniqueness and overrepresentation of FRK class members in Solanaceous species

**Fig. 3:**
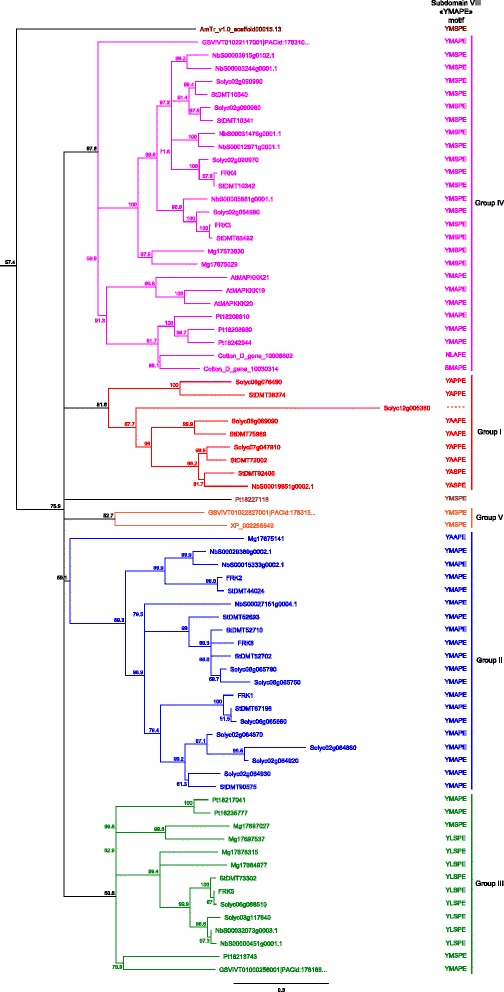
Phylogenetic analysis of the FRK class in representative species of the plant kingdom. Phylogenetic relationships between all the members of the FRK class in *A. thaliana*, *S. chacoense*, *S. lycopersicum*, *S. tuberosum*, *N. benthamiana*, *P. trichocarpa*, *G. raimondtii*, *V. vinifera*, and *A. trichopoda*. Groups are color coded in the tree. Gene IDs and locus tags can be found in Additional file 2: Table S2

In order to ascertain if the expansion of the FRK class is unique to the *Solanaceae*, a family inside the asterid clade, we screened the available genomes of various species spanning the plant kingdom. From the eudicots, the genomes of *Mimulus guttatus*, an asterid in the *Phrymaceae* family, and three other members of the rosid clade, *Populus trichocarpa*, *Vitis vinifera* and *Gossypium raimondii,* were mined to assemble the full compendium of MEKKs. In addition to the above-mentioned dicots species, the genomes of two monocots (*Oryza sativa* and *Zea mays*), an ancient angiosperm (*Amborella trichopoda*, the unique representent of the *Angiospermea* sister clade), two members of the *Gymnospermea* (*Picea glauca* and *Picea abies*), a tracheophyta (*Selaginella mollendorffii*), a moss (*Physcomitrella patens*), and a green algae (*Chlamydomonas reinhardtii*) were also screened. The resulting MEKK phylogenetic tree is shown in Additional file 2: Figure S2, while a close-up of the FRK class is shown in Fig. 3. The FRK class is clearly separate from the rest of the MEKKs, still forming a monophyletic group. Interestingly, no FRK were retrieved from the genomes of the green alga *C. reinhardtii*, the moss *P. patens*, the tracheophyta *S. mollendorffii*, the gymnosperms *P. glauca* and *P. abies* and from the monocots *O. sativa* and *Z. mays*. Furthermore, only one MEKK from the basal angiosperm *A. trichopoda* could be classified as a FRK class member, standing at the base of the FRK clade (represented in brown). The FRK class can now be divided into 5 groups: groups I (red) and II (blue) comprised only solanaceous species members (except one from *M. guttatus* in group II); group III (green), the most heterogeneous group with a low bootstrap value, included proteins from all dicot species; and group IV (magenta), containing proteins from all dicot species, including AtMAPKKK19 to 21. In addition, a fifth group (light orange) appeared, containing only two *V. vinifera* proteins and possibly one FRK-like MEKK from poplar, Pt18227118.

All FRK class members, regardless of the species, harbored the tyrosine residue in the YMAPE motif of subdomain VIII. When considering all species, the various subdomain VIII motifs were similarly spread as observed for the five groups in the solanaceous tree (Fig. 2), with three exceptions: one *M. guttatus* MEKK at the base of group II contained a YAAPE motif; group III, the most diversified group, not only contained the YLSPE motif, but also the YMSPE and YMAPE motifs and; in group IV, the two FRK-like proteins harbored NLAPE and SMAPE motifs, which is totally different from any other MEKKs (Y, W or F). The only *A. trichopoda* FRK-like protein (AmTr_v1.0_scaffold00015.13) harbored a YMSPE motif. MEKKs harboring a YMAPE motifs outside the FRKs are scarce with four in *N. benthamiana*, two in *P. trichocarpa*, three in *M. guttatus*, and two in *P. patens* (see Additional file 2: Figure S2). No other MEKKs from any other species were found to contain the tyrosine residue in the subdomain VIII motif.

**Fig. 4:**
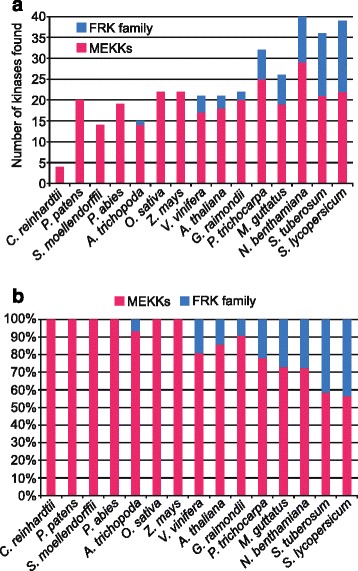
Preponderance of the FRK class members among the MEKKs from various species. **a** Absolute number of FRK class members (*blue*) and number of other MEKKs (*pink*). **b** Percentage of the FRK class members (*blue*) among the MEKK subfamily (*pink*)

Table 1 summarizes the total MEKK numbers, number of FRK members among those MEKKs, genome size and the approximate number of genes for each species. Apart from the single-cell green alga *C. reinhardtii* where only 4 MEKKs were retrieved from a genome comprising ~17,737 genes, land plants from the embryophyta onward, here exemplified by the moss *P. patens*, have roughly five fold or more MEKKs with the exception of *P. glauca* where only 5 MEKKs could be identified from its enormous and complex genome estimated at 20,800 Mbp. Most of the proteins found were either incomplete or had frameshifts, complicating the assembly and analysis of the white spruce MEKK subfamily. Considering this, the *Picea abies* database was also queried. From a genome size of 20,000 Mbp, similar to one from *P. glauca*, 19 different MEKKs including 9 partial sequences were retrieved in *P. abies*. A phylogenetic tree showing the MEKKs from *A. thaliana*, *P. glauca* and *P. abies* is shown in Additional file 2: Figure S3. The first occurrence of a FRK class kinase was in the *Angiospermae*, where the basal angiosperm *A. trichopoda* harbored one FRK out of 15 MEKKs. Interestingly, the two monocots, maize and rice, did not harbor any FRK from their 22 MEKKs. In the eight dicots analyzed, the number of MEKKs varied by 2-fold, ranging from 21 to 40, while the number of FRKs among the MEKKs varied from 2 to 17, up to an 8-fold difference. Figure 4 summarizes the number of MEKKs found in the 15 species examined compared to the FRK class members. From Table 1, genome size in all those species varied from 119 Mbp to 20,800 Mbp and is around 900 Mbp in the solanaceous species, except for *N. benthamiana* that is expected to be around 3500 Mbp. Regardless of genome size, solanaceous species displayed the largest MEKK subfamily. This is particularly true with the FRK class, with first signs of expansion in asterid species (27 %, 7/26 in *M. guttatus*), with a peak in solanaceous species where around 40 % of all MEKKS are part of the FRK class (Fig. 4b). Taken together, these data show a global expansion of the whole MEKK subfamily, especially within the FRK class in solanaceous species.

**Table 1:** Number of MEKKs and FRKs, genome size and expected number of expressed genes for all the species

Species	Number of MEKKs	Number of FRKs	Genome size (Mbp)	Number of expressed genes	References
*C. reinhardtii*	4	0	111	17,737	Phytozome
*P. patens*	20	0	473	26,610	Phytozome
*S. moellendorffii*	14	0	212.5	22,273	Phytozome
*P. glauca*	5	0	20,800	>40,000	Dendrome Genome Project, [26]
*P. abies*	19	0	20,000	28,354	Spruce Genome Project, [68]
*A. trichopoda*	15	1	748	26,846	Amborella Genome Project
*O. sativa*	22	0	372	39,049	Phytozome
*Z. mays*	22	0	3233	39,475	Gramene, EnsemblGenomes
*V. vinifera*	21	4	487	26,346	Phytozome
*A. thaliana*	21	3	135	27,416	Phytozome
*G. raimondii*	22	2	820	40,976	[69]
*P. trichocarpa*	32	7	422.9	41,335	Phytozome, [61]
*M. guttatus*	26	7	312.7	28,140	Phytozome
*N. benthamiana*	40	11	3500	N/A	Sol Genomics Network
*S. lycopersicum*	39	17	950	35,961	Sol Genomics Network
*S. tuberosum*	36	15	840	35,250	Sol Genomics Network

References: Phytozome (http://phytozome.jgi.doe.gov/); Dendrome Genome Project (http://dendrome.ucdavis.edu); Birol et al., 2013 [26]; Amborella Genome Project (http://www.amborella.org); Gramene, EnsemblGenomes (http://ensembl.gramene.org/); Wang et al., (2012) [69]; Tuksan et al., 2006 [61]; Sol Genomics Network (https://solgenomics.net); Spruce Genome Project (http://congenie.org/); and Nystedt et al., 2013 [68]

### FRKs motifs and signatures

The presence of a tyrosine in the subdomain VIII MAPE motif (YMAPE) is the most obvious distinctive feature of the whole FRK class, but is not the only one. In order to uncover other FRK specific signatures, sequence conservation levels from the 12 kinase subdomains were compared using a sequence logo graphical representation. In *A. thaliana*, the FRKs orthologs, AtMAPKKK19-21, are closely related to the AtMAPKKK15-18, and AtMAPKKK15-21 are related to AtMAPKKK13-14 (see Fig. 1). Considering this association, we compared sequence logos from the FRKs alignment, the AtMAPKKK15-18 orthologs alignment, the AtMAPKKK13-14 orthologs alignment and from the AtMAPKKK1-12 orthologs alignment from all relevant species (Additional file 2: Figure S4). Changes that can be observed between the FRKs and the three other sequence logos are either spontaneous change from one amino acid to another as observed for the proline at position 2 (P_2_) in subdomain II or, more frequently, a gradual transition where an underrepresented amino acid in one logo becomes more predominant in the other logos, as observed for R_3_ in subdomain I, G_1_ and C_5_ in subdomain VIb and many others. As for the YMAPE motif and other positions where changes led to more or less specific signatures, changing them one by one or as a whole did not alter the position of the modified FRKs in the phylogeny. Thus, specific alterations in the FRKs kinase subdomain sequences are not sufficient to change the phylogeny, suggesting that sequences outside the 12 subdomains are also involved in FRKs specificity.

### Group I from the FRK class shows a faster evolution rate

Sequence alignments comparison between the four different groups from the FRK class revealed that sequences from group I are more divergent than sequences from others groups. In order to verify if group I evolved more rapidly than the others, we calculated the dN/dS ratios (ω) under two different models (Table 2). The first model (ω_b_) evaluated the background ratio of the whole family, assuming all members evolved at the same rate. The second model (ω_b_, ω_grI_) evaluated the background and group I ratios separately, assuming that group I is evolving at a different rate. The likelihood ratio test (LRT) was done using the log likelihood values of the two models and the group I branch-specific ratio model was largely accepted (*p* = 0.000028). Taken together, these results show that the FRK class group I is unique to the solanaceous species, and it evolved 1.5 time more rapidly (ω_grI_ 0.2165) than groups II to IV (ω_b_ 0.1382).

**Table 2:** Evolutionary rate assessment of group I FRKs as determined by the log likelihood values and parameter estimates under the two models analyzed

Model	p^a^	lnL^b^	ω_b_^c^	ω_grI_^d^
ω_b_ (for all)	138	−18915.804837	0.1471	-
ω_b_, ω_grI_	139	−18907.030984*	0.1382	0.2165

*LRT statistically significant (*p* = 0.000028)

^a^Number of parameters

^b^Log likelihood values

^c^Background dN/dS ratio

^d^Group I dN/dS ratio (if two ratios are estimated)

### The FRK class members are expressed in reproductive tissues

Studied members of the *FRK class* in *S. chacoense* were previously shown to be tightly regulated and preferentially expressed in reproductive tissues. Indeed, *ScFRK1* is expressed in ovules (synergids and egg cell) at anthesis, and its expression decreases rapidly after pollination. *ScFRK2* is weakly expressed in most tissues, but is slightly more expressed in stamen and style, and transiently peaks in ovary immediately following fertilization [46]. *ScFRK3* is expressed in developing ovaries as well as in stamens (Daigle et al., unpublished results). Down-regulation of either those three genes also show reduction of fruit size and seed set due to defects in male and female gametophyte development [46]. This suggests a specialization of the FRK class members in reproductive development. As a first step to support this, we screened databases to determine if *FRK class* members showed preferential expression in reproductive tissues. In *A. thaliana*, both *MAPKKK19* and *20* are highly and specifically expressed in pollen, while no data are available for *MAPKKK21*, absent from the ATH1 chip. In *S. chacoense*, RT-PCRs of *ScFRK3* to *6* showed that others members of the *FRK class* are expressed in reproductive and non-reproductive tissues at various levels (Additional file 2: Figure S5a). For *P. trichocarpa*, in group IV, one out of three *FRKs* was found within the microarray data (Pt18208810) and is highly expressed in both female and male catkins as well as in roots (Additional file 2: Figure S5b). Of the four others *P. trichocarpa* FRKs, three were found in the microarray data (Pt18227118, Pt18213743 and Pt18217041) and are highly expressed in male catkins and moderately expressed in female catkins, while Pt18217041, in group III, is ubiquitously expressed (Additional file 2: Figure S5b). In *S. lycopersicum*, of the 17 FRKs, five from group II were not represented in any available expression database (Solyc02g064870, Solyc02g064880, Solyc02g064920, Solyc02g064930 and Solyc07g047910). Of the remaining 12 *FRKs*, members from group I are mostly expressed in flower buds and slightly in young fruits; members from group II are expressed in reproductive tissues and also in leaves; members from group IV are also expressed mostly in reproductive tissues and with lesser levels in roots and leaves; while members from group III are mostly ubiquitously expressed (Additional file 2: Figure S5c). In *S. tuberosum*, from the 15 *FRKs*, 10 were found in expression databases. The two group I members have different expression patterns; PGSC0003DMG400014807 (St38374) being mostly expressed in somatic tissues while PGSC0003DMG400029548 (St75969) is only expressed inside the fruit. As for tomato, members from group II and IV are expressed in reproductive tissues (immature and mature fruits) and some somatic tissues (stolon for group II and tuber and stolon for group IV). The only member from group III is mostly expressed ubiquitously, as the ones in tomato (Additional file 2: Figure S5d). These results show that many members of the FRK class, among the four species analyzed *(P. trichocarpa*, *S. chacoense*, *S. lycopersicum* and *S. tuberosum*) are preferentially expressed in reproductive tissues, especially in groups I and II. Taken together and considering the reproductive defects observed in *Scfrk1*, *Scfrk2* and *Scfrk3* mutant plants, these results suggest that members of the *FRK class* may have a more specific role in cellular signaling events during plant reproductive development.

## Discussion

### Evolution of the MEKK and the FRK families

The MAPKKK family comprises 80 members in *A. thaliana*, of which 21 are members of the MEKK subfamily (https://www.arabidopsis.org/browse/genefamily/MAPKKK.jsp). Similarly, 22 MEKKs were identified in rice [33], maize [31] and in the diploid cotton *G. raimondii* [52] among the 75, 74 and 78 MAPKKKs identified, respectively. A recent study in tomato identified 89 MAPKKKs with 33 of them classified as MEKKs [53]. Our study extends the knowledge on MAPKKKs with the analysis of 10 other species, from algae to flowering plants. As shown in Fig. 4 and Table 1, from the embryophyta *P. patens* to the rosid *A. thaliana*, the number of the MEKK subfamily members ranged from 14 to 22, except for the gymnosperm *P. glauca*, most probably due to assembly problems arising from its large genome size, although the current number of expressed genes is more or less the same as for other plant species [54]. In the green alga *C. reinhardtii*, only four MEKKs were found. This can be expected considering the unicellular nature of this organism. As a comparison, yeast also has only four MEKKs [55–58]. Genomic analyses of *C. reinhardtii* also reported that of its 1226 gene families, only 26 families harbor 10 or more members and these do not include the MAPK family [59]. The number of MEKKs is higher in the poplar genome, with 32 MEKKs. This can be partly explained by the complexity of its genome, with over 40,000 protein-coding genes, a high level of somatic mosaicism [60] and numerous genome duplications, including the duplication that gave rise to the divergence between the poplar lineage from *Arabidopsis* over 100 million years ago (Ma) [61].

With a total of 26, the number of MEKKs is increasing in the asterid *M. guttatus*, but it reaches the highest number within the *Solanaceae* family, with 40, 39 and 36 MEKKs for *N. benthamiana*, *S. lycopersicum* and *S. tuberosum*, respectively. As shown in Table 1, their genome might be slightly more complex, but their *loci* number is similar to the one from other species. Furthermore, unlike *Brassicaceae*, the *Solanaceae* family has mostly evolved in the absence of polyploidization, except for the cultivated potato and tobacco that are recent polyploids. For instance, tomato, wild potatoes, eggplant and pepper have the same chromosome number architecture [62]. The fact that the MEKK subfamily is much larger in solanaceaous species is probably due to specific duplications of those genes and further evolution that led to novel roles in those species.

The size of the FRK class across the different species of the plant kingdom follows mostly the same pattern than the MEKK subfamily. Indeed, from the *Algae* to the *Gymnospermae*, no FRK class member was found. The first FRK-like kinase member is found in the basal *Angiospermae A. trichopoda* while none is found in rice and maize, suggesting an early loss of the FRKs in monocots. In poplar, *A. thaliana* and *M. guttatus*, 7, 3 and 7 FRK class members have been found, respectively. Again, it is in the *Solanaceae* family that the largest FRK class is found and this holds true when the FRKs ratio is taken into account. Indeed, 28 to 44 % of MEKKs are members of the FRK class in the solanaceous species compared to 0 to 22 % for the others species and 27 % for *M. guttatus*. Clearly, the predominance of the FRK class in solanaceous species suggests the emergence of specific functions unique to those species.

### What makes a FRK?

As observed in Additional file 2: Figure S4, some amino acid sequence alterations are visible in the FRK class when compared to the other MEKKs orthologs. Indeed, most of the amino acid transitions appear in the AtMAPKKK13-14 orthologs, then become more frequent in the AtMAPKKK15-18 orthologs, to finally become unique or almost unique in the FRKs (such as the A_18_ of subdomain I, the S_9_ in subdomain II, the G_1_ and C_5_ in subdomain VIb, or the A_6_ and G_17_ of subdomain IX). This can be explained by recent duplications of the AtMAPKKK13-14 and AtMAPKKK15-18 orthologs and their evolution under specific conditions, which gave rise to the FRK class. New genes tend to be smaller and have a simpler structure (less introns/exons) than older genes [63]. For example in *A. thaliana*, AtMAPKKK13 to 21 are the smallest of the MEKKs family as well as the only one without introns in their gene structure [16]. This supports the idea of a relatively recent origin of the FRK class.

**Fig. 5:**
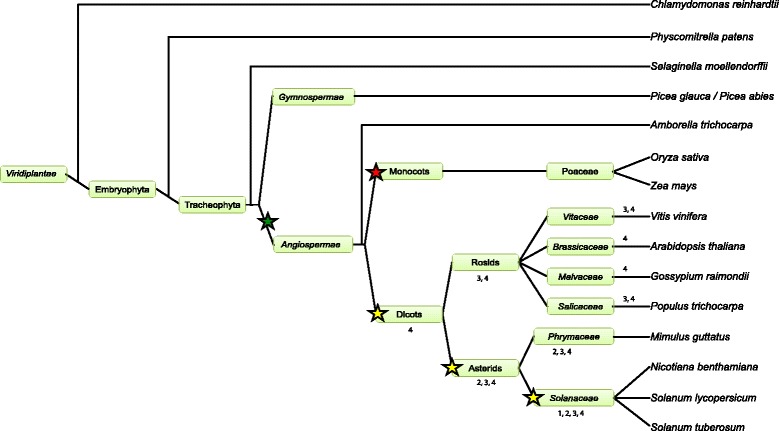
Evolution of the FRK class through the plant kingdom. All species analyzed are represented. The green star shows the first appearance of a FRK-like gene in the genome of *A. trichopoda*. Because of the challenging task of genome sequence assembly in Gymnosperms, it cannot be determined with certainty if the first FRK-like gene appeared before or after *Angiospermae* diversification. The red star shows the loss of the FRK class in monocots while the yellow stars refer to the expansion and emergence of new FRK groups. Numbers refer to FRK groups (1 to 4) found in each species

The first FRK-like protein seems to appear within the *Angiospermae* or might have originated in the common ancestor between the *Angiospermae* and *Gymnospermae,* considering the lack of information from the *Gymnospermae* (Fig. 5). It has rapidly duplicated and diverged in dicots (Fig. 5, yellow stars), creating groups III and IV (groups III and IV are present in all dicots species analyzed) while being rapidly lost in the monocots (Fig. 5, red star). Duplications occurred again within the asterids (group II) and especially within the *Solanaceae* family (Fig. 5, yellow stars), from which group I originated. Although being the most recent, group I is also the most divergent with 49.1 % pairwise identity compared to 53.8, 57.8 and 61.6 % for group II, III and IV, respectively. Group I kinase subdomain sequence logos are also the most variable, showing lesser conservation than groups II-IV. With a ω_gr1_ 1.6 times greater than others groups (ω_b_), the most recent group, group I, is evolving more rapidly. This suggests that this group, which is unique to the *Solanaceae*, is under conditions of higher selective pressure. The slight dN/dS ratios (0.1382 for groups II to IV and 0.2165 for group I) indicate that these proteins are evolving at a relatively slow rate, which is normal considering the need to conserve their catalytic activity towards specific targets, e. g. MKKs. Studies on human kinase proteins also showed low evolutionary rates [64]. When available, access to the genomes of other ancient angiosperms like water lily (*Nymphaeaceae* family) or *Magnolia* (*Magnoliaceae* family) as well as close species from other Solanales families such as *Ipomea batatas* (*Convolvulaceae* family), should give us better resolution of the FRK class evolution.

### The role of the FRK class through evolution of the plant kingdom

The fact that there is one primitive FRK-like MEKK in *A. trichopoda*, and that it has been duplicated in other *Angiospermae* (except for the lost in monocots) should be investigated in terms of gametophyte developmental processes. The FRK class appearance in the *Angiospermae* matches the arrival of the three mitotic divisions of the functional megaspore and cellular specialization, to achieve embryo sac (female gametophyte) maturation [65, 66]. This is consistent with the phenotypes observed in the three FRKs that have been characterized, with a block in mitosis steps, with the vast majority being halted before the first mitosis [46, 47]. In *Tracheophyta* and *Gymnospermae*, one egg cell takes place inside the archegonium within the female gametophyte. Although the number of archegonia found inside an ovule can be highly variable, ranging from 1 to 25 [66], mitotic divisions and cellular specialization is totally different from what is found in the *Angiospermae*. In *Amborella*, the embryo sac follows the same development pattern than most other *Angiospermae* species, with the exception of one more mitotic division of one of the two synergids, producing a third synergid and an egg cell [67]. Further analyses with more species genomes may be an interesting avenue for this research. Moreover, the roles of the FRKs may not be specific to reproduction since .most of them are also expressed in other tissues. Investigations using mutants from species other than *S. chacoense* should also lead to a better understanding of this MAPKKK family and help revealing their roles.

## Conclusion

In *S. chacoense*, three MEKKs, ScFRK1, ScFRK2 and ScFRK3, are involved in reproductive development. Until now, these we thought to be the direct orthologs to the AtMAPKKK19 to 21. Using a deep transcriptomic sequencing of *S. chacoense* ovules and pollen tubes, and the genomes and transcriptomes of 15 other species, the current analysis sheds light on the evolution of the MEKK subfamily, more specifically, the FRK class. Beginning with only one member in *A. trichopoda*, the FRK class expanded during *Angiospermae* evolution, most probably through gene duplication followed by diversification, reaching a maximum in the lineage leading to solanaceous species, where it reached more than 15 members in some cases. The predominance of the FRK class in solanaceous species, representing more than 40 % of the whole MEKK subfamily, strongly suggests the acquisition of new specific roles in these species.

## Availability of supporting data

Contigs assembly can be downloaded from the NCBI Shotgun Assembly Sequences: Genome (WGS) and Transcriptome (TSA) at the following URL: http://www.ncbi.nlm.nih.gov/Traces/wgs/?val=GDZX01.

## Additional files

Additional file 1: Table S1. Protein sequences of the 21 ScMEKKs found in *S. chacoense* ovule and pollen tube transcriptome. **Table S2.** Genomic data resources used to retrieve MEKK subfamily members. **Table S3.** Gene IDs and locus tags (if necessary) of all the MEKKs found. **Table S4.** Primers used for RT-PCRs. (DOCX 50 kb) Additional file 2: Figure S1. Phylogenetic analysis of the MEKK subfamily in four Solanaceous species (*S. chacoense*, *S. tuberosum*, *S. lycopersicum*, and *N. benthamiana*). The kinase domain of the *A. thaliana* BRI1 receptor kinase was used as the outgroup to root the tree. **Figure S2.** Phylogenetic analysis of the whole MEKK subfamily into the 15 studied species and *S. chacoense*. The kinase domain of the *A. thaliana* BRI1 receptor kinase was used as the outgroup to root the tree. **Figure S3.** Phylogenetic analysis of the whole MEKK subfamily in *P. abies*, *P. glauca* and *A. thaliana*. Sequence from AtMPK1 was used as the outgroup to root the tree. **Figure S4.** Sequence logos of the 12 subdomains of the kinase catalytic domain from all FRKs orthologs, AtMAPKKK15-18 orthologs, AtMAPKKK13-14 orthologs, and AtMAPKKK1-12 orthologs. Sequence alignments of all AtMAPKKK1-12 orthologs were used to create a sequence logo using the WebLogo website (http://weblogo.berkeley.edu/logo.cgi) in order to compare the 12 kinase subdomains. The same procedure was applied to AtMAPKKK13-14 orthologs, the AtMAPKKK15-18 orthologs and all the FRK-like proteins, separately. Sequence logos were also used to compare each group (groups I to IV) of the FRK class. **Figure S5.** Expression analysis of members of the *FRK class* in four species. A. RT-PCR expression analyses of *ScFRK*1 to *6* from various tissues in *S. chacoense*. B-D. Absolute expression data taken from *P. trichocarpa* (B), *S. lycopersicum* (C), and *S. tuberosum* (D) available in The Bio-Analytic Resource (BAR) for plant biology, (http://bbc.botany.utoronto.ca). Columns in the heat maps are independant from each other. Lowest expression level in represented in yellow while the highest is in red. (ZIP 11579 kb)

## Abbreviations

MAPKKK, MKKK: Mitogen-activated protein kinase kinase kinase
FRK: Fertilization-related kinase
PK: Protein kinase
MKK: Mitogen-activated protein kinase kinase
MAPK, MPK: Mitogen-activated protein kinase
MW: Molecular weight
HMM: Hidden markov model
LRT: Likelihood-ratio test
