# Supplementary material for: Genome-wide analysis of MAPKKKs shows expansion and evolution of a new MEKK class involved in solanaceous species sexual reproduction
Source: BMC Genomics. 2015 Dec 9;16:1037. doi: 10.1186/s12864-015-2228-3 (PMC4673785; doi:10.1186/s12864-015-2228-3)
Supplement: Additional file 1: Table S1. — Protein sequences of the 21 ScMEKKs found in S. chacoense ovule and pollen tube transcriptome. Table S2. Genomic data resources used to retrieve MEKK subfamily members. Table S3. Gene IDs and locus tags (if necessary) of all the MEKKs found. Table S4. Primers used for RT-PCRs. (DOCX 50 kb) [file 12864_2015_2228_MOESM1_ESM.docx]

**Table S1: Protein sequences of the 21 ScMEKKs found in *S. chacoense* ovule and pollen tube transcriptome.**

|  | Sequence (in amino acids) |
| --- | --- |
| FRK1 | MEKNQEAEIMWKRGRTLGQGGFGFVSLACTTHSDDSPLIPSLIAVKSCMLSHSESLEDEIEFLRMCQDCPHVIRSFGVKVTQEDDILLYNLLLEYASAGSLADRLLNNDQLGLPEFQVQKHTKNVLLGLRFIHRKGIIHCDIKPHNILLTSTDDDDAEEVAKIADFGLSLTLEQSWTQKQGMRGTKRYMAPESLLKQEYGPEADIWALGCTVYELITGTPLWESSNSDPEFDDVLHRIMYEEPNLENDKLSTEAKDFMSYCLIKNPKSRWSAGLLLNHSFLKSADSVLPPKKRKRQHGYMSRLQRRKKAFRTQPHIRHLVIDL* |
| FRK2 | MASIRWSRGRTIGKGAEGTVTLATTHNNFSIAVKSSLFSCSKSLQKEREFLNEFQDCSQIIRCFGADVTEEDNNILYNILLEYAIGGSLAHRIGKKSGLPDFEVKKYSKPILLGLRVVHGKGFVHGDIKPHNILLVGTEKRAKIADFGFASKVGIGSKKRKLRGTPMYMAPESVLDDEYGTPADIWAFGCTVFEMITGKKVWDCTGINDPLHLLCKIGMQSPDLHDDKMSKQAEDFLNKCVDRDPHSRWTADLLLNHPFLSSDNNDVHQRKRKELNLVDLCNSTELKPMKLLTNKRRRKLVERC* |
| FRK3 | MDWVRGEAVGHGSFGKVNLAIPRKQSTQFLPLMVMKSSGVSHSVSLVNEKKILDELNGCPEIIRCFGESYSFENGDSLYNLLLEYATGGSLAEKLKKSENYKLSEFEVSGYTKGILKGLQYVHEVGYAHCDIKLQNILLGEDGKVKIADFGLAKRVDDELRYELRGTPLYMSPEMVTGGEQGTPADIWALGCVVAEMVAGVPAFRFSNVTELLMAIGVKNQLPQIPEELSEEGKDFLSKCFVQDPRKRWTAEMLLKHPFVADHDTVTLLNEEIKNGVPSISPKCPFDFPDWVSDESAQSSVTCSITFLPSPENLNSSCGRWSTSPAERLMGLVSEFSTESEWCSHDDWVTVR* |
| FRK4 | MDWVRGETVGHGSFGKVSFAIPRNQSTLFSPSMVVKSSSASCSATLMNEKMILDELKGCPQIINCVGDSYSYENGEKLYNVLLEYACGGALSDKLNNSGDQRLPEFEVRKYTKGLIKGIHYIHKNGYVHCDIKLPNILLGKNGQVKISDFGLSKRAESKKYDKLRCELRGTPLYMSPEMVIGGEQDTPADIWALGCVVAEMATGNPVWRCSDITKLLMTIGLGDQLPEIPQNLSEEGKDFLEKCLVKDPKKRWTAEMLLKHPFVADEDDTVLLNEERCNSGSPSTSPRCPFDFPDWVSNKSAESSVTCSITSLPSPAFQESMNWSDESWSTSPTERIRELVCECRPEFEWSTADGWVSVR* |
| FRK5 | MQQGMESKKKIEVEELKNVNEFGDGVSWYRGAMLGKGSFGYVYLANLKNPKSKNRYLPSVMAVKSAEVSVSGSIQKEREVLSNIKGCPYVIRCFGDETTSGNNGVMAYNLLLEYGSGGTLAERINKLGLAEFEVRLYTRSMLRGLNHIHAIGYVHCDMKPENVLLVPNSSKGSVEFRAKIGDLGLAKRENQSKKRRLETYWRGTPMYLSPEAVADNVQESPADIWALGCIVLEMLTGKPPWDRKDAEDVLKKIGEGHELPEIPGDLSKEAKDFLKGCFVRKPTYRWTAEMLLIHPFVEGLCDNDDGVEERQEVEDINEVDSMQLVTETDDEVSILKKIGAAFRRGIRWYWSEDDTEITEDEIASSFAEERMSIRSSSIDSGFNSMIDTSSQVASRNPSNNSSKCPLKFTIPAGVLALGGRREI* |
| FRK6 | MAGLLWKRGTTLGQGGFGVVSLASTSNALFRSVTLPSLIALKSCNYSASQSLKEEVEILRMFKHSPYIAHCFGANVSFEDNVNLYNSLPEYASGGSLADRLQNCNSLSESEVKKHTKNVLLGLSCIHNNGIIHCDIKYGNILLVGRDKTAKIADFGLSVTLEQGMNQKQGVIRGTERYMAPESVINTEYTSQVDIWALGCTVHELITGTPMWEDADGDDVLDKIEFEEPKFQNSKLSNEAQNFLEKCLVKNPSTRWTADMLLNHTFLQNSSKVANTAKTRKKKSDSMSLLHKPIQKITFKIDHHKFSRQLLDPKPLLDKPIKKITFKFGNHKFSRQLLDPKPLLHKPIQKITFKIGNHKFMRQSPDLKEVENEPCGRVLGTDNR |
| isotig10269 | MSRQMANAAFHKSKTLDNKYMLGDEIGKGAYGRVYKGLDLENGDFVAIKQVSLENIAQEDLNVIMQEIDLLKNLNHKNIVKYLGSLKTKSHLFIILEYVENGSLANIVKPNKFGPFPESLVAVYISQVLEGLVYLHEQGVIHRDIKGANILTTKEGLVKLADFGVATKLTEADVNTHSVVGTPYWMAPEVIEMSGVCAASDIWSVGCTVIELLTCLPPYYDLQPMPALFRIVQDDHPPIPDSLSPAITDFLRQCFKKDARQRPDAKTLLSHPWIQNSRRALQSSLRHSGTIRNIEEDGSAVREASNEDDKGAAGSSSSDKAKESSTTLASPEVLETSKSEEVDRSSSIRIEERTDKIEDQFTSDPVPTLAIHEKSPIQHNADGLAVNKESALQSSTDLGEPDKVFANGELEFSESRGGNTVGRKVEEKGHGVNVYSASSSSGQKNTDYSPRKAVKTSVVPQGNELSRFSDPPGDASLDDLFHPLEKNLENRAAEVSLSASSSQIAQNNAIAETGKNDLATKLRATIAKKQMESESGPPNGGDLLSIMMGVLKEDVIDMDGLGFDDKLPTENLFHLQAVEFSKLVSSLRTDESEDVIVSACQKLIAFFHQRPDQKLVFVTQHGLLPLMELLEVPKTRVMCSVLQVLNLIVQDNTDSQENACLVGLIPVVMSFAAPDRPREIRMEAAYFFQQLCQSSPLTLQMFIANRGIPVLVGFLEADYAKYREMVHMAIDGMWQVFKLQRSTSRNDFCRIAAKNGILLRLINTLYSLNEAARLASASGGGGFPPDGLAPRPRSGPLDPGNSSFMQTEMPSYGTDQPDMLKIKNGDRVLPSGMQEPSRTSASHSPDSPFFRQDGERPRSSNATMEASGLSRLPDGNLVTKDRESLDRYKNDLFRAEIDLRQQRGGNTSRISTDRGSKQMEGGSYGFPASTASQQENVRPLLSLLEKEPPSRHFSGQLEYHNLPGLEKHESILPLLHASNEKKTNGLDFLMAEFAEVSGRGRENTNLESLPRSPHKAATKKVGGAASTDGIASTSGFASQTASGVLSGSGVLNARPGSAASSGILSHMAPPWNADVAREYLEKVADLLLEFSAADTTVKSYMCSQSLLSRLFQMFNKIEPPILLKLLKCINHLSTDPHCLENLQRADAIKYLIPNLDLKEGPLVSQIHHEVLNALFNLCKINKRRQEQAAENGIIPHLMHFIMTSSPLKQYALPLLCDMAHASRNSREQLRAHGGLDVYLSLLEDELWSVTALDSIAVCLAHDNESRKVEQALLKKDAIQKMVKFFECCPEQHFLHILEPFLKIITKSSRINTTLAVNGLTPLLVSRLDHRDAIARLNLLKLIKAVYEHHPRPKQLIVENDLPQKLQNLIEERRDGQTSGGQVLVKQMATSLLKALHINTVL |
| isotig14157 | MPSWWKSSKEAKKKPTKESFIDSLHRKFKSPAEVKSPGKSGGSRRHSSDIASEKGSLSQAQSRASSPSKHVSRCQSFAERPMAQPLPLPGVRPANVGRLDSGISPSAKSRVEKASKPSLLPLPKPACIRHRLDPADTDGELVFASISSECSIESDDPIDSRQRSPLATDYEAGSRIAAGSPSSLVVKDQSAVGQISLKETTRPVSLSPSRNVSSVSPKRRPLSSHVTTLQVPPPGAFCSAPDSSMSSPSRSPMRAAASEQVTSSTLWAGRAYPDLPSLGSGHCSSPGSGQNSGHNSMGGDMSGQLFWQPCRGSPEYSPIPSPRMTSPGPSSRIHSGAVTPIHPRAVGGAAELQTSWPDDGKAQSHPLPLPPLTISNSSPFSHSNSVATSPSVPRSPGRAENLASPGSRWKKGKLLGRGTFGHVYVGFNSDSGEMCAMKEVTLFSDDAKSKESAKQLAQEIALLSRLRHPNIVQYYGTETVGDKLYIYLEYVSGGSIYKLLQEYGAFGEAAIRSYTHQILSGLAYLHAKNTVHRDIKGANILVDPNGRIKLADFGMAKHITGQSCPLSFKGSPYWMAPEVIKNSSGCNLAVDIWSLGCTVLEMATSKPPFSQYEGVAAMFKIGNSKELPTIPEQLSDEAKDFVRKCLQREPRLRPTAAQLLDHPFVKNVATLEKPNISPAPADPPCAGANGVKSLGIGQTRNIPTSESERLATHSSRVSKSNFHCRSFLLIILYFLTYLIFIEKLSKESI |
| isotig15589 | MPSWWGKSKAKKKATKESFIDSLHRKFKSPAEAKSPSKSGGSRRHNNDIASEKGSQSQAQSRSSSPSKNVSRCQSFAESALAQPLPLPGLPSASVVRADSGISQSAKPRVEKGSKSSLFLPLPKPACIRHRLDPADADGELVFASISSECSVESDDPTDSRQRSPLAFDYETGNRTPLGSPPRLAVKDQSAVGQISIKEATEPVNLSPSGHVSSRSPKRRPLNSHLSSIQIPSHGALCSAPDSSISSPSRSPMRAAGCEQVSSSTFWAGKTYPDLPLLGSGHCSSPGSGQNSGHNSMGGDMVGQLFWHPSRGSPEYSPIPSPRMTSPGPSSRIHSGAVTPIHPRAGGGASELQTNWPDDTKPESHPLPRPPLAISNSSPFSHSNSVATSPSVPRSPGRAENLSSPGSRWKKGKLLGRGTFGHVFVGFNSDSGEMCAMKEVTLFSDDAKSKESAKQLTQEISLLSRLRHPNIVQYYGSEMVPDKLYIYLEYVSGGSIYKLLQEYGPFGETAIRSYTQQILSGLAYLHAKNTVHRDIKGANILVDPNGRIKLADFGMAKHITGQSCPLSFKGSPYWMAPEVIKHTSGCNLAVDVWSLGCTVLEMATSKPPWSQYEGVAAMFKIGNGKELPAIPEELSDEGKDFVRKCLQREPRNRPTAAELLEHPFVKDAAPPEKPNMFPTSFDLPCAAANGIKPLVVGSARNYPTPDSERLAIHSSRASKSKFHCSDILIPKNISCPVSPIGSPLPRSPHNLNGRMSPSPISSPLNTSGSSTPISGGNGAIPFRHINQSVYLQEARTVPNSPYMNGSSYWDPDVLRGSPSGSHAFQELASVEYDALGKQFGRLATGELCNGQSALANRVSQQLLRDHVKSISSVDLNPCPPLGGRTGGT |
| isotig24879 | MYAKQKKLRPRLDRTNALKNVDYDASQSVPSTPSSLRDQPAHRTRSLDLYPVPDRTSFRIDGAAGEFDTICRSLGLSPEDFAIPVAAWEAGKPCSRSDRLRSTRLSDDRRDSDIKLEDANELSDSVRTAARVTVDAESNIRLNNLPENVIEVTISESDVETECSHSDCFGSEDELETVDEVRNGVRGIVGGKLKHILYSSPENLIKVRVSESEDDNLPTDVKCGIKGFRPPRLAPPATDVDDFTSAWDFIKSFGPADDEGMVSPLPDESTSDDILVNEQVEEIAKNEERREEFVRNASQVSESSSEISTDRDNDSSVSRTENDGACEKPFEQGVRDAAESSKSPSGDSYSLISKTSSKSPTGESDDVISEMRSKSLSGDSYALILKPVHSVSPNGSPSIKSWQKGDFLGSGSFGTVYEGFTDDGFFFAVKEVSLIDPGNQQSLFQLEQEISLLSRFRHRNIVRYHGTNKDESKLYIFLELVTKGSLASVYRKYRLRDSHVSDYTRQILSGLHYLHSREVMHRDIKCANILVDANGSVKLADFGLAKATQLNNIKSCKGTAFWMAPEVVNRKSNGYGTPADIWSLGCTVLEMLTGQIPYSHLEGMQALFRIGRGEPPPIPDTLSTEAQDFIKSCLRVNPNDRPTAAELLEHPFVMKPPSNFSGPLAP |
| isotig27346 | MIVRSSLDSHVIDVNSNGSRCGGGGSQCSGSGRVIDLGSKLIKEDICRYSDSSKASDVIQLQVQSVELNYDRGGVVCAARVISDVSGAGTRLDDGVTVNGVVSNGNEPVSLLGGGGGGGIRGLRPPLLAPPPVMSLPIVDDACSTWDIFRAFGPEDHRESGIAGHGICRSEVVNGDEEYMKDEEDEENSNRMILGVSSLLSQSSSFTNTSNDDDSSSCTTERMSIISPNGRFTRFITGWDKGGLLGRGSFGSVYEGISHDGFFFAVKEVSLLDQGDGGRQSLYQLEQEIELLRQFEHENIVQYYGTDKNDSKLYIFLELVTQGSLLNLYQKYHLRDSQVSAYTRQILHGLKYLHDRNVVHRDIKCANILVHANGSVKLADFGLAKATKLNDVKSFKGTALWMAPEVVNRKNPGYGLAADIWSLGCTVLEMLTRQFPYSHLENPMQVLFQIGKGEPPVVPNTLSEDARNFINHCLQVDPSARPTATQLLEHPFVKQTLPSSSGSASPLNLGRRL |
| isotig27662 | MPSLWKAFSLSSSSQHSSSSTTTTASTTSSPADSPNTRRIYGGGRKLTRQRKLRHVSDDDLGLRRPNIQALIIDERSKSLPGSPDSYADFGSGSRSSHHHLRHCSNSSAVPLPLPLPELNSLPKQNSVDSNLPGRVDRELLSPPLARETFGRTPAEVKSSQHPRSSTPTYQRRGFPQDLNAEGVEFRLNVPARSAPSSGFTSPVRSPKRFSTQDLFHHPLHQASSSPSEAYSFQLSPTRVINSADHSPLSSPILPSSANRIRNSRSGAVHSHHKSLPESSLGWNEANNNNVHPLPLPPGVPRQPESCTMHSNMDKPCVSPAKGQWLKGKLLGRGTFGSVYEATNCETGALCAMKEVDLTPDDPKSAECIKQLEQEIRVLQQLKHQNIVQYYGSEVMEDRFCIYLEYVHPGSINKYVREHCGAMTESIVRNFTRHIVSGLAYLHSTKTIHRDIKGANLLVDASGVVKLADFGLAKHLSSCATDLSLKGSPHWMAPEVMQAVLRKDANPELALAVDIWSLGCTVIEMFTGQPPWGELSWVQAMFGVLNKSPPIPEKLSPEGKDFLQCCFRRKPADRPSAIKLLEHAFLRSTSSLEHSVNVAGCSEDSPGKKFHDTLSPKNPINHKKEQKPLLPGTSGRHAKSPCSSETCRQPQPETCEYGAASHHSPRSALVVFPCISSMELNSSSRAASPSSVPSSFRLGPENRSPYRIIGRKFQTSV |
| isotig33548 | MHSWWGKSSSKDVRRKSTKESFIDIINRKLKIFTTEKSSGKSGSSRRQRKDTNSVKGSQSRVSRSPSPSTPDSRSQVFADRTSSQPLPLPEGHSSNVHLVDSDNSASIILVTGEVSESSLTLPPPMPRHLPHGPAAAGVDRDLPTASVSCDSSSDSDDLTDSRLLSPQTSDYENGSRTALNSPSSLKQKVQSPIASNASSGEMVKSATLLSNNQAISTSPRQRLLSSHVPGLQIPHHGASYSAPDSSMSSPSRSPMRVFGHETVMNSGFWLGKPHGEITFLGSGHCSSPGSGQNSGHNSIGGDMSAQPFWPHSRCSPECSPVPSPRMTSPGPGSRIHSGAVTPLHPRAGGTLTESSTASLDNGKQQSHRLPLPPISIPHSSVFSLSCSMAPAIPRSPGRTGNPPSPGPRWKKGRLIGRGTFGHVYRGFNSESGEMCAMKEVTLFSDDPKSRESAQQLGQEISLLSRLRHPNIVQYYGSETVDDKLYIYLEYVSGGSIYKILQEYGQLGELAIQSYTQQILSGLAYLHAKNTVHRDIKGANILVDPNGRIKLADFGMAKHITGQYCPLSFKGSPYWMAPEVIKNSNGCNLAVDIWSLGCTVLEMATTKPPWSQYEGVAAIFKIGNSKEVPAIPYHLSDEGKDFVRQCLQRNPLHRPTASQLLKHPFVKSTAPMERFIGIGHLKDPPCVGSEEVAVHHQPRDSNFFPGFSDVPVPRSCPVSPVGIESPIYHSQSPKHMSGRLSPSTISSPRAVSGSSTPLSGGGGAVPLSNPMMSTTSSSEDVGTSPKTQSCFYPDDYTSHGLKSDMFRETLPYGNGFFGENFGGHAQSGVNGQPYQGQSVLANRVAQQLLRDQVKLSPSFDLNPGSPVFSWDNGV |
| isotig33816 | GWCSIPWEIIKPRCSKKEKEHLGSRLSRKTPQKLHVADRVSDIRISAPISAPTTPYASPGLSPLKAGDLLNHNYMAFPGAFQVCSAPEMPPSDRLQYPGFSYHVLPEKNAFSVDNSPHHSPRVSPQRSRKIASGPASPLHQLLPNENSTARRESSAQGNVHPLPLPPLGATPSHSTSIPPVPSNAELTPIKGQWQKGKLIGRGTFGSVYVASNRETGALCAMKEVELLPDDPKSAESIRQLEQEINVLSHLKHPNIVQYYGSEIVGDRFYIYLEYVHPGSINKFIRDHCETITESIVRNFTRHILCGLAYLHSKKTIHRDIKGANLLVDAYGVVKLADFGMAKHLNGQAANLSLKGSPYWMAPELLQSVMQTDTTTDLAFATDIWSLGCTVIEMLNGRPPWSEYEAAAAMFKVLKDTPPIPETLSPEGKDFLRCCFCRNPAERPSASMLLEHRFMRVSHQPDVPSFIKPVGVIRVKEKLNSQKEQTTYNLDQGRLSLER |
| isotig34208 | MPAWWGKKSTKNKDPQVKEKEREKYVKPRSFDELLGRNSPRTSKDFSGSGSGFSGFDSGSSLEKAHPLPKPSVSSLGNDHGVVLGCGSVSVSSTSSSGSSDGGGAVNTDQAQLDTFRGLGDNRLSPLSRSPVRSRGTTTTSSPLHPRFSSLNLDSPTGKLDDVRSECHQLPLPPGSPPSPSTLPNPRTCVVAEGATINMSKWKKGRLLGRGTFGHVYLGFNRENGQMCAIKEVKVVSDDQTSKECLKQLNQEIILLSNLTHPNIVRYHGSELDEETLSVYLEYVSGGSIHKLLQEYGPFREPVIQNYTRQILSGLSFLHARNTVHRDIKGANILVDPNGEIKLADFGMAKHITSSASVLSFKGSPYWMAPEVVMNTSSYGLAVDIWSLGCTILEMATSKPPWSQYEGVAAIFKIGNSKDFPEIPEHLSNDAKSFIRSCLQREPSTRPTASQLLEHPFVKNQSTAKVAHVGVTKESYPRSFDGSRTPPVLELHPGGRNISPGRNISPAEGNYASHPVITVSRPLICTRESVKAITSLPVSPTSSPLRQYEPARKSCYLSPPHPSYGIGGQSGYEANDYSMFQARPSTRTTLEPWLEIPQFRAQTPSRSPRTRPIL |
| isotig34351 | MIGCGAFGRVYMGMNVDSGELLAIKEVSIAMNGASRERAQAHVRELEEEVNLLKNLSHPNIVRYLGTAREVGSLNILLEFVPGGSISSLLGKFGSFPESVIRMYTKQLLLGLEYLHKNGIMHRDIKGANILVDNKGCIKLADFGASKKVVELATMTGAKSMKGTPYWMAPEVILQTGHSFSADIWSVGCTIIEMATGKPPWSQQYQEVAALFHIGTTKSHPPIPEHLSAEAKDFLLKCLQKEPHLRTSASNLLQHPFVTGEHQEARPFLRSSFLGNPENMAAQRMDVRTSTNPDIRATCNGLKDVCDVSTVRCSTLYPENSLGNESLWKIGNSDDDMCQMDNDDFMFGASVKCSSDLHSRANYKSFNPMSEPDNDWPCKFDESPELTKSQANLDFAQATIKPTNSPIMSYKEDAFAFSGGQSAAEDDDELTESKIRAFLDEKAMDLKKLQSPLYEEFLNSMNVSSTPSPAGIGNKENFPSNVNLPPKSRSPKRFLSRRLSTATEGACAPSPVTHSKRISNIGGLGNEAIQELQSPRNNEWKDPLGSQRDTVNSSFSERQRKWKEELDEELQRKREIMRQAVNLSPPMDPVLNRCRSKSRFASPGR |
| isotig36119 | MIGCGAFGQVYMGMNLDSGELLAVKQVMIAANSASKEKAQSHVKELEEEVKLLKNLSHQHIVRYLGIVREEDTLNILLEFVPGGSISSLLGKFGSFPEPVIRTYTKQLLLGLDYLHKNGIMHRDIKGANILVDNKGCIKLADFGASKKVVELATISGAKSMKGTPYWMSPEVIRQTGHSFSADIWSVGCTVIEMTTGKPPWSQQYQEVAALFYIVGTTKAHPPIPEHVSVEAKDFLLKCLQKEPELRPSASELLQHPFVTGEAQLSLPDGSSSMMGKAQGHSYSSGHNAKSVAGSVDICNLGTLNISTENTDNLSEARNMWRGNSSDDDMCQIDDNDNLLLDGGTTFSTVKTLDDFNKSFNPIAEPSDDWNCDYGMTPQSRQGNTDLVNNQEGGLGAGSSASPNNNSAVLCGPSISEDEDELTESKIRAFLDEKALELKKLQTPLYEEFYNSLNPSYSSPQLVEATIDETTPNYLRLPPKSRSPSRGPIGSPSTGIDIITSPSPGSSNRRTSCIGSGSN |
| isotig37024 | MAEFSKCSCLKPTKWLKGKVIGSGSFGSVHLAMDKATGGFFVLKSTDSEAGFKCLENEVEILENLDSPHIVKFIGKDLSFEANGKRKLSLFLEFMTGGSLADVAEKFGGSLDEEVICLYTKGILKGLKCLHESGIVHCDLKCKNILLGTSGEIKLADLGCAKRIKDHKVKGITKSLSKSIGGTPLWMAPEILRNEELDFAADIWSLGCTIIEMATGKTPWGGDICTNPLAAVLKIACSNEMPQFPSHFSDVGLDFLIKCLERDSKKRWKVEQLLDHPFVSKGKSVKIKNFKVASTPASVLDSGIFSEMDFSDDQLSSDEDESTSGNALRDHGFWISTREGKMDLESSESWINVRN |
| isotig37044 | TSSPRNYKKNRVNKPKSFDESIFIPKNSPTSFATNYGYYSYTYTQDKKLHPVFPLPLPLDSPNFSTRSSSGSSPTSSQFNDDEEQGISPLFSPFRNKCNDWSRTSNESSKPNSPCTSPTTYPVELDSPNGKQEECNHPRHPLPLPPNSSTKGRTGQWKKGKLLAKGTFGNVYAGFNSNNGQMCAIKEVRIIFNDTTSKERLKQLNQEITLLSQFSHPNIVQYYGSELKGDKLSLYLEYVPGGSILKLLQEYGPFEEQIISSYTRKILSGLVFLHERNIAHRDIKGATILVNAKGEIKLADFGMAKHINSCCLMDSFKGSPYWMAPEVVKDAGGFSVAVDIWSLGCTVVEMATAKPPYEGADGKDSPEIPRNLSDNAKSFLKLCLQRNPSHRLTAVQLLHHPFVQA |
| isotig38767 | MFMEYVQGGTLSDLIKKQGGALDESMIKLYAQQILQGLDYLHSIGIVHCDVKGQNILIGENGDIKIADLGCAKLLRDEKNSGFSGTPAFMAPEVARGEEQGFAADIWAFGCTIIEMATGSVPWSEIKDPVSALFRIGYSGDLPQFPNNLSNDAREFLGKCLMKCPNERWTANQLLQHPFLQSVESNSWKFEELKRDSPTSILDQGFWNSFEVMESSSLESTNTVDSATDRIRQLIGNVGISCSLMPNWVEEDDWVTVRCNDTEENSIISEPNCEMIDGFGELLDMEISESIVFSEDEFVTLLNLEALLVD |
| isotig41825 | VVSVAKSRFSDEVFAVKSVELSESQLLQKEQIILSQLSSSYVVSYKGYDVTKEKDKLMFNLMMEYMPDGTLSDEIQKQGGRMNERLIGYYTKQIVQGLDYLHSRSIAHCDLKGQNILVGKTGAKIADFGCARWIDPVEREGNAEPIGGTPMFMAPEVARGEEQGCPADIWGLGCTIIEMATGGSPWTNVTNAASLLYKIAFSGQSPEIPKSSLYKQGIS |

Table S2: Genomic data resources used to retrieve MEKK family members.

| **Species** | **Data assembly, version or reference** | **URL** |
| --- | --- | --- |
| *Solanum tuberosum* | Peptides from Assembly v3.4 (PGSC_DM_v3.4_pep) | <http://solgenomics.net/organism/Solanum_tuberosum/genome> |
| *Solanum lycopersicum* | Peptides from Assembly 2.40 were used (Solanum_lycopersicum.SL2.20.17.pep.all) | <http://mips.helmholtz-muenchen.de/plant/tomato/download/index.jsp> |
| *Nicotiana benthamiana* | Peptides from Assembly v0.4.4 (Niben.genome.v0.4.4.proteins.annotated) | <ftp://ftp.solgenomics.net/genomes/Nicotiana_benthamiana/annotation/> |
| *Mimulus guttatus* | CDS from Assembly v1.1 (Mguttatus_140_cds) | <ftp://ftp.jgi-psf.org/pub/compgen/phytozome/v9.0/Mguttatus_v1.1/>) |
| *Populus trichocarpa* | CDS from Assembly v8.0 (Ptrichocarpa_156_cds) | <http://www.plantgdb.org/XGDB/phplib/download.php?GDB=Pt> |
| *Amborella trichopoda* | Peptides from EVM 27 (Amborella EVM 27 predicted proteins) | <http://www.amborella.org/> |
| *Oryza sativa* | *In Silico* analysis as described by Rao *et al*. (2010) [1] |  |
| *Zea mays* | Data from Kong *et al*. (2013) [2] |  |
| *Picea glauca* | Unigenes contigs from TreeGenes (unigene_Picea glauca) | <http://dendrome.ucdavis.edu/treegenes/transcriptome/transcr_summary.php> |
| *Picea abies* | Peptides from the Spruce Genome Project (congenie.org) | http://congenie.org/start |
| *Physcomitrella patens* | Peptides from the V6 version (P.patens.V6_filtered_cossmoss_protein_codons) | <http://www.plantgdb.org/XGDB/phplib/download.php?GDB=Pp> |
| *Chlamydomonas reinhardtii* | Proteins from the Creinhardtii_236 version (Creinhardtii_236_proteins) | <ftp://ftp.jgi-psf.org/pub/compgen/phytozome/v9.0/Creinhardtii/annotation> |
| *Selaginella mollendorffii* | Proteins from the version 9 (Smoellendorffii_91_protein) | <ftp://ftp.jgi-psf.org/pub/compgen/phytozome/v9.0/Smoellendorffii> |
| *Vitis vinifera* | Proteins from version 9 (Vvinifera_145_protein) | <ftp://ftp.jgi-psf.org/pub/compgen/phytozome/v9.0/Vvinifera> |
| *Gossympim Raimondii* | Data from Yin et al. (2013) [3] |  |

**References**

1. Rao KP, Richa T, Kumar K, Raghuram B, Sinha AK: **In Silico Analysis Reveals 75 Members of Mitogen-Activated Protein Kinase Kinase Kinase Gene Family in Rice**. *DNA Research* 2010, **17**(3):139-153.

2. Kong F, Wang J, Cheng L, Liu S, Wu J, Peng Z, Lu G: **Genome-wide analysis of the mitogen-activated protein kinase gene family in Solanum lycopersicum**. *Gene* 2012, **499**(1):108-120.

3. Yin Z, Wang J, Wang D, Fan W, Wang S, Ye W: **The MAPKKK Gene Family in Gossypium raimondii: Genome-Wide Identification, Classification and Expression Analysis**. *International Journal of Molecular Sciences* 2013, **14**(9):18740-18757.

Table S3: Gene IDs and locus tags (if necessary) of all the MEKKs found

| **Species** | **MEKKs** | **Acc. Number NCBI** | **Other (known) names** |
| --- | --- | --- | --- |
| *A. thaliana* | AtMAPKKK1 | Gene ID: 837421 AT1G09000 | ANP1, NP1, NPK1-related protein kinase 1 |
|  | AtMAPKKK2 | Gene ID: 841937 AT1G54960 | ANP2, NP2, NPK1-related protein kinase 2 |
|  | AtMAPKKK3 | Gene ID: 841792 AT1G53570 |  |
|  | AtMAPKKK4 | Gene ID: 842674 AT1G63700 | YDA, YODA |
|  | AtMAPKKK5 | Gene ID: 836819 AT5G66850 |  |
|  | AtMAPKKK6 | Gene ID: 819989 AT3G07980 | MAP3Kε2 |
|  | AtMAPKKK7 | Gene ID: 820555 AT3G13530 | MAP3Kε1 |
|  | AtMAPKKK8 | Gene ID: 826409 AT4G08500 | MEKK1 |
|  | AtMAPKKK9 | Gene ID: 826407 AT4G08480 | MEKK2 |
|  | AtMAPKKK10 | Gene ID: 826406 AT4G08470 |  |
|  | AtMAPKKK11 | Gene ID: 826810 AT4G12020 |  |
|  | AtMAPKKK12 | Gene ID: 819774 AT3G06030 | ANP3, NP3, NPK1-related protein kinase 3 |
|  | AtMAPKKK13 | Gene ID: 837226 AT1G07150 |  |
|  | AtMAPKKK14 | Gene ID: 817555 AT2G30040 |  |
|  | AtMAPKKK15 | Gene ID: 835600 AT5G55090 |  |
|  | AtMAPKKK16 | Gene ID: 828796 AT4G26890 |  |
|  | AtMAPKKK17 | Gene ID: 817812 AT2G32510 |  |
|  | AtMAPKKK18 | Gene ID: 839324 AT1G05100 |  |
|  | AtMAPKKK19 | Gene ID: 836843 AT5G67080 |  |
|  | AtMAPKKK20 | Gene ID: 824193 AT3G50310 |  |
|  | AtMAPKKK21 | Gene ID: 3770588 AT4G36950 |  |
| *S. lycopersicum* | Solyc01g079750 |  | SlMAPKKK4 |
|  | Solyc01g098980 |  | SlMAPKKK8 |
|  | Solyc01g103240 |  | SlMAPKKK9 |
|  | Solyc01g104530 |  | SlMAPKKK10 |
|  | Solyc02g064870 |  |  |
|  | Solyc02g064880 |  |  |
|  | Solyc02g064920 |  |  |
|  | Solyc02g064930 |  | SlMAPKKK13 |
|  | Solyc02g064980 |  | SlMAPKKK14 |
|  | Solyc02g065110 |  | SlMAPKKK15 |
|  | Solyc02g090430 |  | SlMAPKKK20 |
|  | Solyc02g090970 |  | SlMAPKKK21 |
|  | Solyc02g090980 |  | SlMAPKKK22 |
|  | Solyc02g090990 |  | SlMAPKKK23 |
|  | Solyc03g025360 |  | SlMAPKKK26 |
|  | Solyc03g117640 |  | SlMAPKKK29 |
|  | Solyc04g064590 |  | SlMAPKKK33 |
|  | Solyc04g079400 |  | SlMAPKKK35 |
|  | Solyc06g036080 |  | SlMAPKKK37 |
|  | Solyc06g065660 |  |  |
|  | Solyc06g065750 |  |  |
|  | Solyc06g065790 |  |  |
|  | Solyc06g068510 |  | SlMAPKKK38 |
|  | Solyc07g047910 |  | SlMAPKKK48 |
|  | Solyc07g051860 |  | SlMAPKKK50 |
|  | Solyc07g051870 |  | SlMAPKKK51 |
|  | Solyc07g051880 |  | SlMAPKKK52 |
|  | Solyc07g051890 |  | SlMAPKKK53 |
|  | Solyc07g051920 |  | SlMAPKKK54 |
|  | Solyc07g051930 |  | SlMAPKKK55 |
|  | Solyc07g053170 |  | SlMAPKKK56 |
|  | Solyc07g064820 |  | SlMAPKKK59 |
|  | Solyc08g069090 |  | SlMAPKKK63 |
|  | Solyc08g076490 |  | SlMAPKKK64 |
|  | Solyc08g081210 |  | SlMAPKKK66 |
|  | Solyc11g006000 |  | SlMAPKKK80 |
|  | Solyc11g033270 |  | SlMAPKKK82 |
|  | Solyc12g005360 |  | SlMAPKKK83 |
|  | Solyc12g088940 |  | SlMAPKKK87 |
| *S. tuberosum* | StDMT01980 | PGSC0003DMP400001453#PGSC0003DMT400001980 |  |
|  | StDMT01981 | PGSC0003DMP400001454#PGSC0003DMT400001981 |  |
|  | StDMT10340 | PGSC0003DMP400007205#PGSC0003DMT400010340 |  |
|  | StDMT10341 | PGSC0003DMP400007206#PGSC0003DMT400010341 |  |
|  | StDMT10342 | PGSC0003DMP400007207#PGSC0003DMT400010342 |  |
|  | StDMT20760 | PGSC0003DMP400014144#PGSC0003DMT400020760 |  |
|  | StDMT20761 | PGSC0003DMP400014145#PGSC0003DMT400020761 |  |
|  | StDMT20764 | PGSC0003DMP400014148#PGSC0003DMT400020764 |  |
|  | StDMT23236 | PGSC0003DMP400015822#PGSC0003DMT400023236 |  |
|  | StDMT26091 | PGSC0003DMP400017815#PGSC0003DMT400026091 |  |
|  | StDMT26093 | PGSC0003DMP400017816#PGSC0003DMT400026093 |  |
|  | StDMT31805 | PGSC0003DMP400021570#PGSC0003DMT400031805 |  |
|  | StDMT36857 | PGSC0003DMP400025003#PGSC0003DMT400036857 |  |
|  | StDMT38374 | PGSC0003DMP400026063#PGSC0003DMT400038374 |  |
|  | StDMT38860 | PGSC0003DMP400026412#PGSC0003DMT400038860 |  |
|  | StDMT38861 | PGSC0003DMP400026413#PGSC0003DMT400038861 |  |
|  | StDMT38863 | PGSC0003DMP400026414#PGSC0003DMT400038863 |  |
|  | StDMT38864 | PGSC0003DMP400026415#PGSC0003DMT400038864 |  |
|  | StDMT39934 | PGSC0003DMP400027063#PGSC0003DMT400039934 |  |
|  | StDMT39935 | PGSC0003DMP400027064#PGSC0003DMT400039935 |  |
|  | StDMT39936 | PGSC0003DMP400027065#PGSC0003DMT400039936 |  |
|  | StDMT39937 | PGSC0003DMP400027066#PGSC0003DMT400039937 |  |
|  | StDMT40869 | PGSC0003DMP400027723#PGSC0003DMT400040869 |  |
|  | StDMT44024 | PGSC0003DMP400029864#PGSC0003DMT400044024 |  |
|  | StDMT44025 | PGSC0003DMP400029865#PGSC0003DMT400044025 |  |
|  | StDMT52693 | PGSC0003DMP400035528#PGSC0003DMT400052693 |  |
|  | StDMT52702 | PGSC0003DMP400035533#PGSC0003DMT400052702 |  |
|  | StDMT52710 | PGSC0003DMP400035535#PGSC0003DMT400052710 |  |
|  | StDMT52743 | PGSC0003DMP400035555#PGSC0003DMT400052743 |  |
|  | StDMT57171 | PGSC0003DMP400038451#PGSC0003DMT400057171 |  |
|  | StDMT61013 | PGSC0003DMP400041077#PGSC0003DMT400061013 |  |
|  | StDMT61014 | PGSC0003DMP400041078#PGSC0003DMT400061014 |  |
|  | StDMT61015 | PGSC0003DMP400041079#PGSC0003DMT400061015 |  |
|  | StDMT63857 | PGSC0003DMP400043019#PGSC0003DMT400063857 |  |
|  | StDMT63858 | PGSC0003DMP400043020#PGSC0003DMT400063858 |  |
|  | StDMT64172 | PGSC0003DMP400043252#PGSC0003DMT400064172 |  |
|  | StDMT65492 | PGSC0003DMP400044175#PGSC0003DMT400065492 |  |
|  | StDMT67126 | PGSC0003DMP400045281#PGSC0003DMT400067126 |  |
|  | StDMT67198 | PGSC0003DMP400045331#PGSC0003DMT400067198 |  |
|  | StDMT67237 | PGSC0003DMP400045362#PGSC0003DMT400067237 |  |
|  | StDMT67238 | PGSC0003DMP400045363#PGSC0003DMT400067238 |  |
|  | StDMT67244 | PGSC0003DMP400045369#PGSC0003DMT400067244 |  |
|  | StDMT72002 | PGSC0003DMP400048685#PGSC0003DMT400072002 |  |
|  | StDMT73302 | PGSC0003DMP400049581#PGSC0003DMT400073302 |  |
|  | StDMT73303 | PGSC0003DMP400049582#PGSC0003DMT400073303 |  |
|  | StDMT73304 | PGSC0003DMP400049583#PGSC0003DMT400073304 |  |
|  | StDMT73788 | PGSC0003DMP400049944#PGSC0003DMT400073788 |  |
|  | StDMT75969 | PGSC0003DMP400051445#PGSC0003DMT400075969 |  |
|  | StDMT83928 | PGSC0003DMP400056168#PGSC0003DMT400083928 |  |
|  | StDMT90575 | PGSC0003DMP400062250#PGSC0003DMT400090575 |  |
|  | StDMT90976 | PGSC0003DMP400062651#PGSC0003DMT400090976 |  |
|  | StDMT92406 | PGSC0003DMP400064081#PGSC0003DMT400092406 |  |
| *N. benthamiana* | NbS00000157g0020.1 |  |  |
|  | NbS00000451g0001.1 |  |  |
|  | NbS00001100g0032.1 |  |  |
|  | NbS00003244g0001.1 |  |  |
|  | NbS00003915g0102.1 |  |  |
|  | NbS00005060g0004.1 |  |  |
|  | NbS00005088g0102.1 |  |  |
|  | NbS00005088g0110.1 |  |  |
|  | NbS00005881g0001.1 |  |  |
|  | NbS00007599g0011.1 |  |  |
|  | NbS00008390g0005.1 |  |  |
|  | NbS00008866g0001.1 |  |  |
|  | NbS00008984g0009.1 |  |  |
|  | NbS00009115g0011.1 |  |  |
|  | NbS00009351g0002.1 |  |  |
|  | NbS00010186g0025.1 |  |  |
|  | NbS00011174g0009.1 |  |  |
|  | NbS00012971g0001.1 |  |  |
|  | NbS00013054g0005.1 |  |  |
|  | NbS00013254g0023.1 |  |  |
|  | NbS00014457g0014.1 |  |  |
|  | NbS00015333g0002.1 |  |  |
|  | NbS00016417g0008.1 |  |  |
|  | NbS00016734g0008.1 |  |  |
|  | NbS00019856g0007.1 |  |  |
|  | NbS00019951g0002.1 |  |  |
|  | NbS00021069g0005.1 |  |  |
|  | NbS00023343g0001.1 |  |  |
|  | NbS00024903g0006.1 |  |  |
|  | NbS00026683g0007.1 |  |  |
|  | NbS00027151g0004.1 |  |  |
|  | NbS00028218g0004.1 |  |  |
|  | NbS00028906g0101.1 |  |  |
|  | NbS00029389g0002.1 |  |  |
|  | NbS00031476g0001.1 |  |  |
|  | NbS00031960g0003.1 |  |  |
|  | NbS00032073g0003.1 |  |  |
|  | NbS00036784g0001.1 |  |  |
|  | NbS00041747g0015.1 |  |  |
|  | NbS00045519g0007.1 |  |  |
|  | NbS00047703g0009.1 |  |  |
|  | NbS00053762g0005.1 |  |  |
|  | NbS00057060g0004.1 |  |  |
|  | NbS00058641g0004.1 |  |  |
|  | NbS00058681g0001.1 |  |  |
| *M. guttatus* | Mg17669913 | mgv1a019444m\|PACid:17669913 |  |
|  | Mg17670449 | mgv1a025509m\|PACid:17670449 |  |
|  | Mg17673630 | mgv1a008588m\|PACid:17673630 |  |
|  | Mg17675007 | mgv1a021391m\|PACid:17675007 |  |
|  | Mg17675029 | mgv1a009594m\|PACid:17675029 |  |
|  | Mg17675141 | mgv1a022903m\|PACid:17675141 |  |
|  | Mg17676315 | mgv1a021677m\|PACid:17676315 |  |
|  | Mg17676821 | mgv1a021688m\|PACid:17676821 |  |
|  | Mg17677279 | mgv1a003315m\|PACid:17677279 |  |
|  | Mg17677329 | mgv1a025764m\|PACid:17677329 |  |
|  | Mg17677469 | mgv1a020948m\|PACid:17677469 |  |
|  | Mg17677819 | mgv1a024020m\|PACid:17677819 |  |
|  | Mg17682496 | mgv1a003649m\|PACid:17682496 |  |
|  | Mg17682524 | mgv1a026298m\|PACid:17682524 |  |
|  | Mg17682543 | mgv1a003872m\|PACid:17682543 |  |
|  | Mg17683764 | mgv1a023179m\|PACid:17683764 |  |
|  | Mg17684977 | mgv1a008079m\|PACid:17684977 |  |
|  | Mg17685306 | mgv1a001468m\|PACid:17685306 |  |
|  | Mg17687864 | mgv1a019636m\|PACid:17687864 |  |
|  | Mg17687895 | mgv1a017805m\|PACid:17687895 |  |
|  | Mg17688029 | mgv1a006112m\|PACid:17688029 |  |
|  | Mg17689954 | mgv1a002847m\|PACid:17689954 |  |
|  | Mg17689955 | mgv1a002848m\|PACid:17689955 |  |
|  | Mg17689956 | mgv1a002852m\|PACid:17689956 |  |
|  | Mg17691318 | mgv1a002756m\|PACid:17691318 |  |
|  | Mg17691840 | mgv1a002030m\|PACid:17691840 |  |
|  | Mg17692445 | mgv11b019097m\|PACid:17692445 |  |
|  | Mg17693018 | mgv1a025547m\|PACid:17693018 |  |
|  | Mg17695757 | mgv1a001116m\|PACid:17695757 |  |
|  | Mg17696182 | mgv1a025013m\|PACid:17696182 |  |
|  | Mg17697027 | mgv1a017677m\|PACid:17697027 |  |
|  | Mg17697537 | mgv1a023811m\|PACid:17697537 |  |
| *P. trichocarpa* | Pt18206224 | POPTR_0005s14170.1\|PACid:18206224 |  |
|  | Pt18206854 | POPTR_0005s16210.1\|PACid:18206854 |  |
|  | Pt18207901 | POPTR_0005s03640.1\|PACid:18207901 |  |
|  | Pt18208810 | POPTR_0005s18240.1\|PACid:18208810 |  |
|  | Pt18208825 | POPTR_0005s06330.1\|PACid:18208825 |  |
|  | Pt18208980 | POPTR_0005s18230.1\|PACid:18208980 |  |
|  | Pt18208984 | POPTR_0005s09700.1\|PACid:18208984 |  |
|  | Pt18213743 | POPTR_0006s19480.1\|PACid:18213743 |  |
|  | Pt18214134 | POPTR_0021s00300.1\|PACid:18214134 |  |
|  | Pt18216310 | POPTR_0003s12910.1\|PACid:18216310 |  |
|  | Pt18216602 | POPTR_0003s13010.1\|PACid:18216602 |  |
|  | Pt18217041 | POPTR_0003s18240.1\|PACid:18217041 |  |
|  | Pt18221893 | POPTR_0013s02360.1\|PACid:18221893 |  |
|  | Pt18222326 | POPTR_0014s15310.1\|PACid:18222326 |  |
|  | Pt18223733 | POPTR_0014s03480.1\|PACid:18223733 |  |
|  | Pt18224506 | POPTR_0004s00910.1\|PACid:18224506 |  |
|  | Pt18227118 | POPTR_0009s13360.1\|PACid:18227118 |  |
|  | Pt18227472 | POPTR_0009s07730.1\|PACid:18227472 |  |
|  | Pt18230291 | POPTR_0012s12950.1\|PACid:18230291 |  |
|  | Pt18232632 | POPTR_0015s12870.1\|PACid:18232632 |  |
|  | Pt18235201 | POPTR_0001s28520.1\|PACid:18235201 |  |
|  | Pt18235697 | POPTR_0001s09520.1\|PACid:18235697 |  |
|  | Pt18235698 | POPTR_0001s09520.3\|PACid:18235698 |  |
|  | Pt18235699 | POPTR_0001s09520.2\|PACid:18235699 |  |
|  | Pt18235777 | POPTR_0001s03390.1\|PACid:18235777 |  |
|  | Pt18242206 | POPTR_0010s10210.1\|PACid:18242206 |  |
|  | Pt18242572 | POPTR_0007s11420.1\|PACid:18242572 |  |
|  | Pt18242944 | POPTR_0007s10930.1\|PACid:18242944 |  |
|  | Pt18243158 | POPTR_0007s07900.1\|PACid:18243158 |  |
|  | Pt18243224 | POPTR_0007s04070.1\|PACid:18243224 |  |
|  | Pt18244724 | POPTR_0002s13050.1\|PACid:18244724 |  |
|  | Pt18245706 | POPTR_0002s08950.1\|PACid:18245706 |  |
|  | Pt18246774 | POPTR_0002s21350.1\|PACid:18246774 |  |
|  | Pt18248307 | POPTR_0008s14880.1\|PACid:18248307 |  |
|  | Pt18248308 | POPTR_0008s14880.2\|PACid:18248308 |  |
| *V. vinifera* | GSVIVT01000047001\|PACid:17816662 |  |  |
|  | GSVIVT01000256001\|PACid:17816833 |  |  |
|  | GSVIVT01004254001\|PACid:17818962 |  |  |
|  | GSVIVT01007446001\|PACid:17820298 |  |  |
|  | GSVIVT01008938001\|PACid:17821456 |  |  |
|  | GSVIVT01009575001\|PACid:17821956 |  |  |
|  | GSVIVT01012031001\|PACid:17823759 |  |  |
|  | GSVIVT01013909001\|PACid:17825038 |  |  |
|  | GSVIVT01017915001\|PACid:17828009 |  |  |
|  | GSVIVT01019739001\|PACid:17829310 |  |  |
|  | GSVIVT01019796001\|PACid:17829353 |  |  |
|  | GSVIVT01022117001\|PACid:17831030 |  |  |
|  | GSVIVT01022827001\|PACid:17831503 |  |  |
|  | GSVIVT01023048001\|PACid:17831655 |  |  |
|  | GSVIVT01023079001\|PACid:17831673 |  |  |
|  | GSVIVT01023216001\|PACid:17831764 |  |  |
|  | GSVIVT01026487001\|PACid:17834182 |  |  |
|  | GSVIVT01028897001\|PACid:17835891 |  |  |
|  | GSVIVT01030452001\|PACid:17836951 |  |  |
|  | GSVIVT01038760001\|PACid:17842953 |  |  |
|  | XP_002266649 |  |  |
| *G. raimondii* | Cotton_D_gene_10000305 |  |  |
|  | Cotton_D_gene_10001555 |  |  |
|  | Cotton_D_gene_10002230 |  |  |
|  | Cotton_D_gene_10003410 |  |  |
|  | Cotton_D_gene_10006972 |  |  |
|  | Cotton_D_gene_10008602 |  |  |
|  | Cotton_D_gene_10017021 |  |  |
|  | Cotton_D_gene_10018040 |  |  |
|  | Cotton_D_gene_10019751 |  |  |
|  | Cotton_D_gene_10024896 |  |  |
|  | Cotton_D_gene_10025330 |  |  |
|  | Cotton_D_gene_10029669 |  |  |
|  | Cotton_D_gene_10030314 |  |  |
|  | Cotton_D_gene_10030328 |  |  |
|  | Cotton_D_gene_10030510 |  |  |
|  | Cotton_D_gene_10031221 |  |  |
|  | Cotton_D_gene_10032983 |  |  |
|  | Cotton_D_gene_10033856 |  |  |
|  | Cotton_D_gene_10034692 |  |  |
|  | Cotton_D_gene_10038046 |  |  |
|  | Cotton_D_gene_10039321 |  |  |
|  | Cotton_D_gene_10040437 |  |  |
| *O. sativa* | LOC_Os01g50370 |  |  |
|  | LOC_Os01g50400 |  |  |
|  | LOC_Os01g50410 |  |  |
|  | LOC_Os01g50420 |  |  |
|  | LOC_Os02g21700 |  |  |
|  | LOC_Os02g32610 |  |  |
|  | LOC_Os02g35010 |  |  |
|  | LOC_Os02g44642 |  |  |
|  | LOC_Os03g15570 |  |  |
|  | LOC_Os03g18170 |  |  |
|  | LOC_Os03g49640 |  |  |
|  | LOC_Os03g55560 |  |  |
|  | LOC_Os04g35700 |  |  |
|  | LOC_Os04g47240 |  |  |
|  | LOC_Os04g56530 |  |  |
|  | LOC_Os05g46750 |  |  |
|  | LOC_Os05g46760 |  |  |
|  | LOC_Os07g02780 |  |  |
|  | LOC_Os08g32600 |  |  |
|  | LOC_Os09g21510 |  |  |
|  | LOC_Os10g04000 |  |  |
|  | LOC_Os10g04010 |  |  |
| *Z. mays* | AC209208.3-FGT001 |  | ZmMAPKKK6 |
|  | BT034005.1 |  | ZmMAPKKK3 |
|  | GRMZM2G017654-T01 |  | ZmMAPKKK14 |
|  | GRMZM2G034877-T01 |  | ZmMAPKKK8 |
|  | GRMZM2G041774-T01 |  | ZmMAPKKK22 |
|  | GRMZM2G044557-T01 |  | ZmMAPKKK13 |
|  | GRMZM2G064613-T01 |  | ZmMAPKKK15 |
|  | GRMZM2G066120-T01 |  | ZmMAPKKK11 |
|  | GRMZM2G093316-T01 |  | ZmMAPKKK5 |
|  | GRMZM2G098828-T01 |  | ZmMAPKKK16 |
|  | GRMZM2G130927-T01 |  | ZmMAPKKK12 |
|  | GRMZM2G140726-T01 |  | ZmMAPKKK1 |
|  | GRMZM2G156800-T01 |  | ZmMAPKKK9 |
|  | GRMZM2G165099-T01 |  | ZmMAPKKK19 |
|  | GRMZM2G173965-T01 |  | ZmMAPKKK21 |
|  | GRMZM2G175504-T01 |  | ZmMAPKKK4 |
|  | GRMZM2G180555-T01 |  | ZmMAPKKK10 |
|  | GRMZM2G305066-T01 |  | ZmMAPKKK18 |
|  | GRMZM2G439350-T01 |  | ZmMAPKKK17 |
|  | GRMZM2G476477-T01 |  | ZmMAPKKK20 |
|  | GRMZM2G540772-T01 |  | ZmMAPKKK2 |
|  | LOC100281440 precursor_GRMZM2G378479-T01 |  | ZmMAPKKK7 |
| *A. trichopoda* | AmTr_v1.0_scaffold00007.31 |  |  |
|  | AmTr_v1.0_scaffold00007.127 |  |  |
|  | AmTr_v1.0_scaffold00007.128 |  |  |
|  | AmTr_v1.0_scaffold00009.178 |  |  |
|  | AmTr_v1.0_scaffold00015.13 |  |  |
|  | AmTr_v1.0_scaffold00021.129 |  |  |
|  | AmTr_v1.0_scaffold00021.130 |  |  |
|  | AmTr_v1.0_scaffold00024.46 |  |  |
|  | AmTr_v1.0_scaffold00034.46 |  |  |
|  | AmTr_v1.0_scaffold00069.132 |  |  |
|  | AmTr_v1.0_scaffold00071.8 |  |  |
|  | AmTr_v1.0_scaffold00099.42 |  |  |
|  | AmTr_v1.0_scaffold00105.35 |  |  |
|  | AmTr_v1.0_scaffold00106.46 |  |  |
|  | AmTr_v1.0_scaffold00156.23 |  |  |
|  | AmTr_v1.0_scaffold00169.27 |  |  |
| *P. glauca* | gnl\|UG\|Pgl#S48359113 - partial |  |  |
|  | gnl\|UG\|Pgl#S55344276 - partial |  |  |
|  | gnl\|UG\|Pgl#S55346491 - partial |  |  |
|  | gnl\|UG\|Pgl#S55347412 - partial |  |  |
|  | gnl\|UG\|Pgl#S55347734 |  |  |
| *S. moellendorffii* | 24258\|PACid:15417035 |  |  |
|  | 55695\|PACid:15422250 |  |  |
|  | 65764\|PACid:15407835 |  |  |
|  | 73444\|PACid:15406871 |  |  |
|  | 73721\|PACid:15409183 |  |  |
|  | 74353\|PACid:15409107 |  |  |
|  | 76216\|PACid:15414207 |  |  |
|  | 82067\|PACid:15408094 |  |  |
|  | 86240\|PACid:15421274 |  |  |
|  | 113703\|PACid:15418451 |  |  |
|  | 139837\|PACid:15406526 |  |  |
|  | 164743\|PACid:15412823 |  |  |
|  | 438664\|PACid:15422682 |  |  |
|  | 444546\|PACid:15417419 |  |  |
| *P. patens* | Pp1s8_299V6.1 |  |  |
|  | Pp1s10_200V6.1 |  |  |
|  | Pp1s17_36V6.1 |  |  |
|  | Pp1s20_383V6.1 |  |  |
|  | Pp1s35_323V6.1 |  |  |
|  | Pp1s43_178V6.1 |  |  |
|  | Pp1s44_113V6.1 |  |  |
|  | Pp1s66_286V6.1 |  |  |
|  | Pp1s99_12V6.1 |  |  |
|  | Pp1s100_149V6.1 |  |  |
|  | Pp1s131_142V6.1 |  |  |
|  | Pp1s136_5V6.3 |  |  |
|  | Pp1s151_3V6.1 |  |  |
|  | Pp1s151_4V6.1 |  |  |
|  | Pp1s188_1V6.1 |  |  |
|  | Pp1s216_64V6.1 |  |  |
|  | Pp1s281_41V6.1 |  |  |
|  | Pp1s281_44V6.1 |  |  |
|  | Pp1s351_40V6.1 |  |  |
|  | Pp1s387_39V6.1 |  |  |
| *C. reinhardtii* | Cre07.g317300.t1.3\|PACid:27563909 |  |  |
|  | Cre10.g464100.t1.2\|PACid:27579952 |  |  |
|  | Cre17.g733300.t1.3\|PACid:27571481 |  |  |
|  | g396.t1\|PACid:27578203 |  |  |
| *P. abies* | MA_11360g0010 partial |  |  |
|  | MA_12842g0020 partial |  |  |
|  | MA_12842g0030 |  |  |
|  | MA_15568g0010 |  |  |
|  | MA_17530g0010 |  |  |
|  | MA_21577g0010 |  |  |
|  | MA_38298g0010 partial |  |  |
|  | MA_52416g0010 |  |  |
|  | MA_72906g0010 |  |  |
|  | MA_106297g0010 |  |  |
|  | MA_130871g0010 partial |  |  |
|  | MA_183773g0010 partial |  |  |
|  | MA_238866g0010 partial |  |  |
|  | MA_276143g0010 partial |  |  |
|  | MA_10426326g0010 |  |  |
|  | MA_10427758g0010 |  |  |
|  | MA_10431188g0010 partial |  |  |
|  | MA_10431312g0010 |  |  |
|  | MA_10434470g0010 partial |  |  |

Table S4: Primers used for RT-PCRs

| **Primers** | **Sequences** | **Amplicon (bp)** |
| --- | --- | --- |
| FRK1 RT-PCR F | 5’-CCAAAGAAGAGGAAGAGGCAAC-3’ | 350 |
| FRK1 RT-PCR R | 5’-GGTGAGAAATAACCCATACGACG-3’ |  |
| FRK2 RT-PCR F | 5’-GGATTCGCAAGCAAGGTTGG-3’ | 360 |
| FRK2 RT-PCR R | 5’-CCTTCCTCTTCCTCTGATGAAC-3’ |  |
| FRK3 RT-PCR F | 5’-GTCCATTTGATTTCCCAGATTG-3’ | 343 |
| FRK3 RT-PCR R | 5’-GTGGCAACCAATGTTATGTAC-3’ |  |
| FRK4 RT-PCR F | 5’-GCAACAGTGGCAGCCCTTCAAC-3’ | 338 |
| FRK4 RT-PCR R | 5’-TGGCCTAGCCTAATCACTAAATTG-3’ |  |
| FRK5 RT-PCR F | 5’-GAAATCGCCTCTTCGTTTGCTG-3’ | 227 |
| FRK5 RT-PCR R | 5’-CTACAGAGAGTGTAGCCAAATAAG-3’ |  |
| FRK6 RT-PCR F | 5’-GCAATTGCTAGATCCGAAGC-3’ | 288 |
| FRK6 RT-PCR R | 5’-GGATCAAACCCATACATACTTC-3’ |  |
