# Supplementary material for: Genome-wide analysis of MAPKKKs shows expansion and evolution of a new MEKK class involved in solanaceous species sexual reproduction
Source: BMC Genomics. 2015 Dec 9;16:1037. doi: 10.1186/s12864-015-2228-3 (PMC4673785; doi:10.1186/s12864-015-2228-3)

Subdomain I

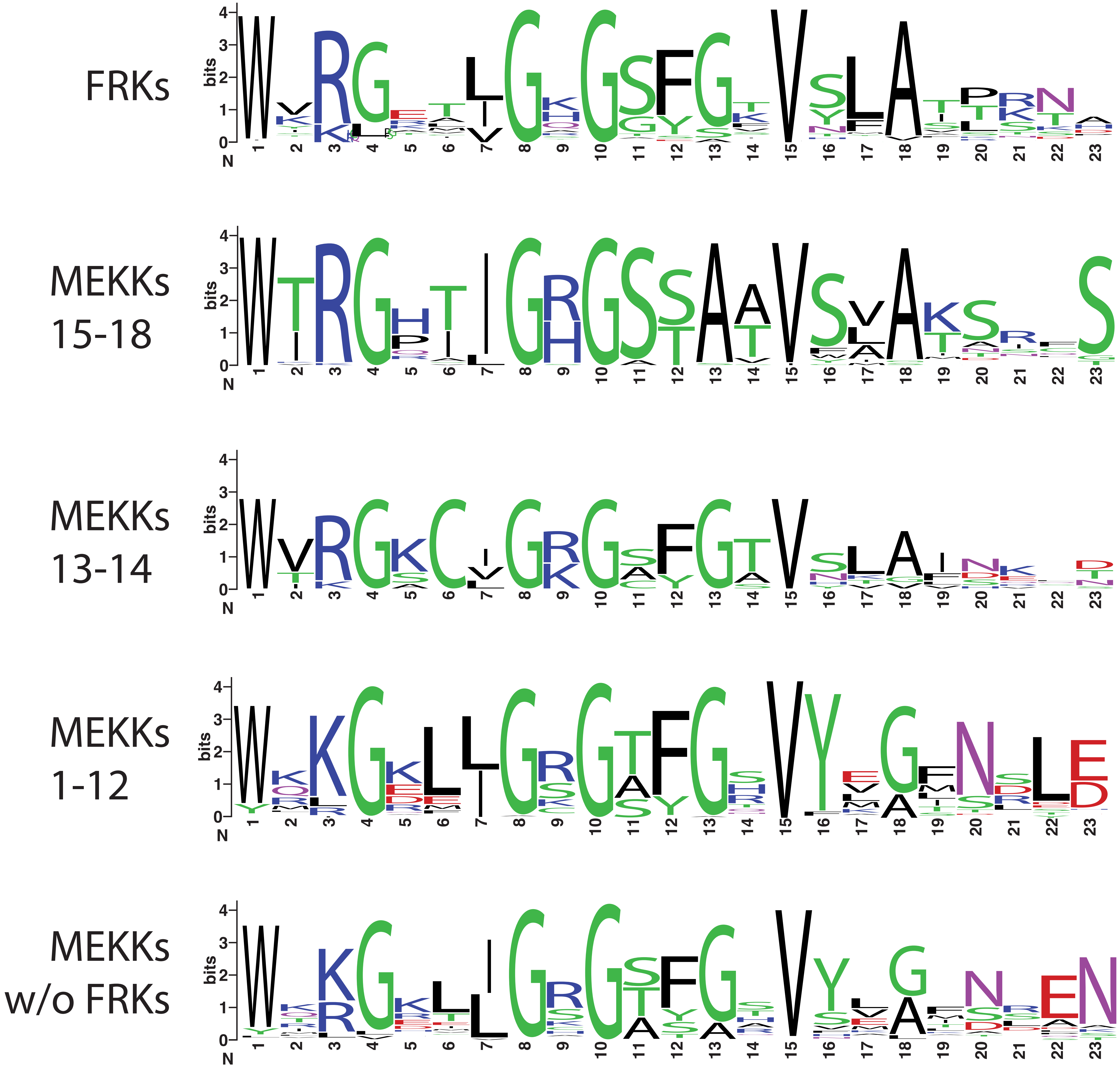

Subdomain II

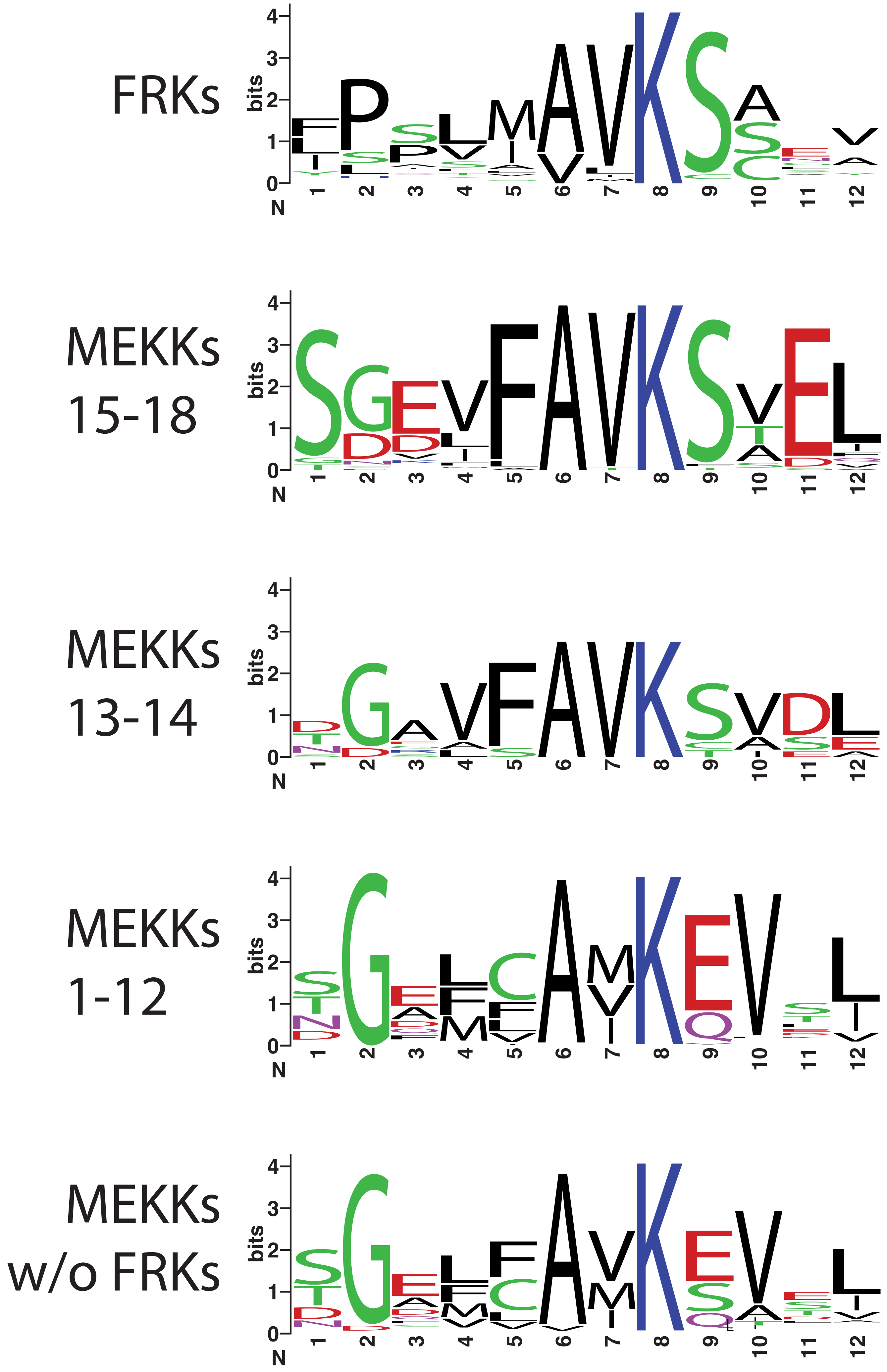

Subdomain III

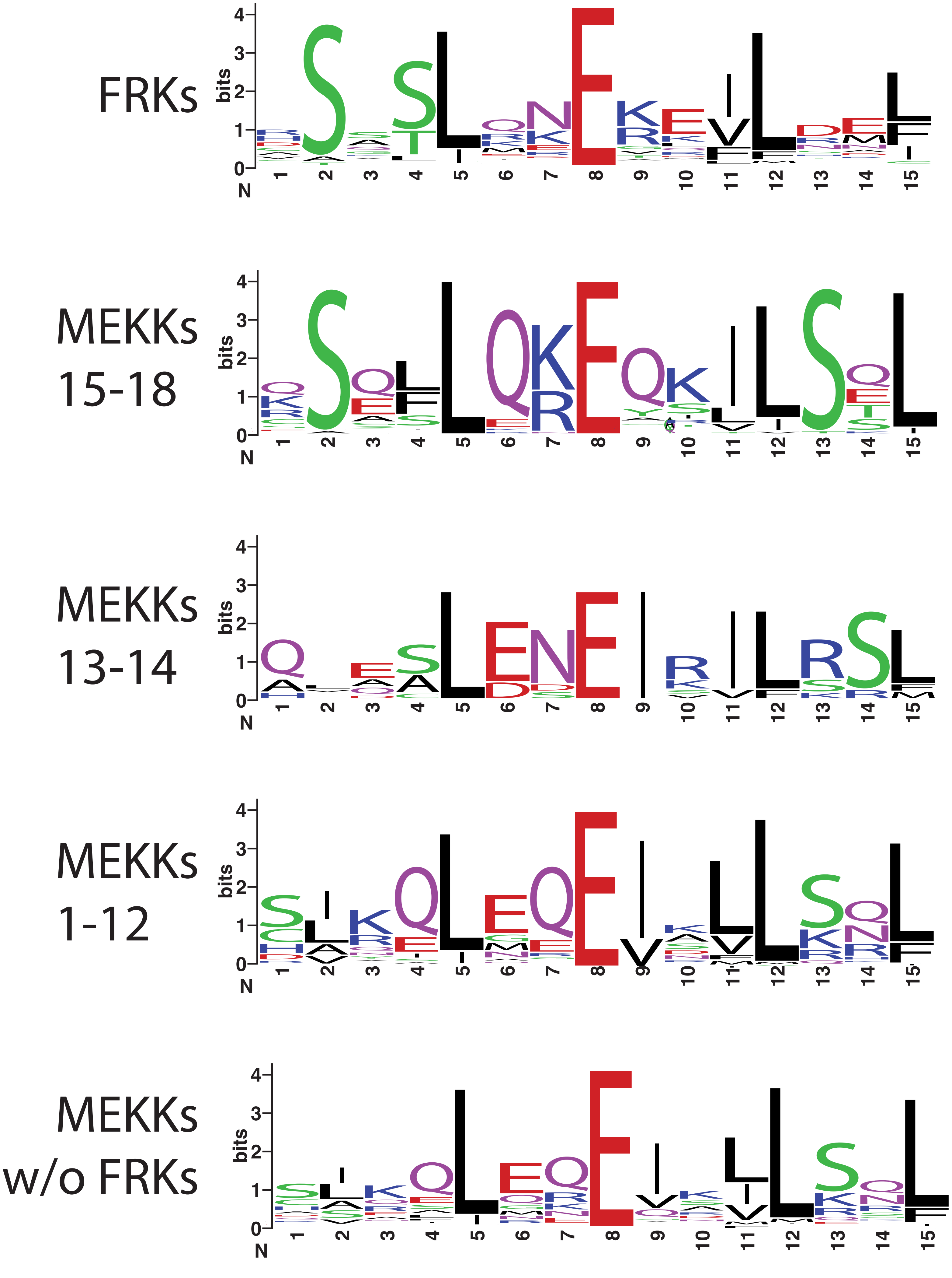

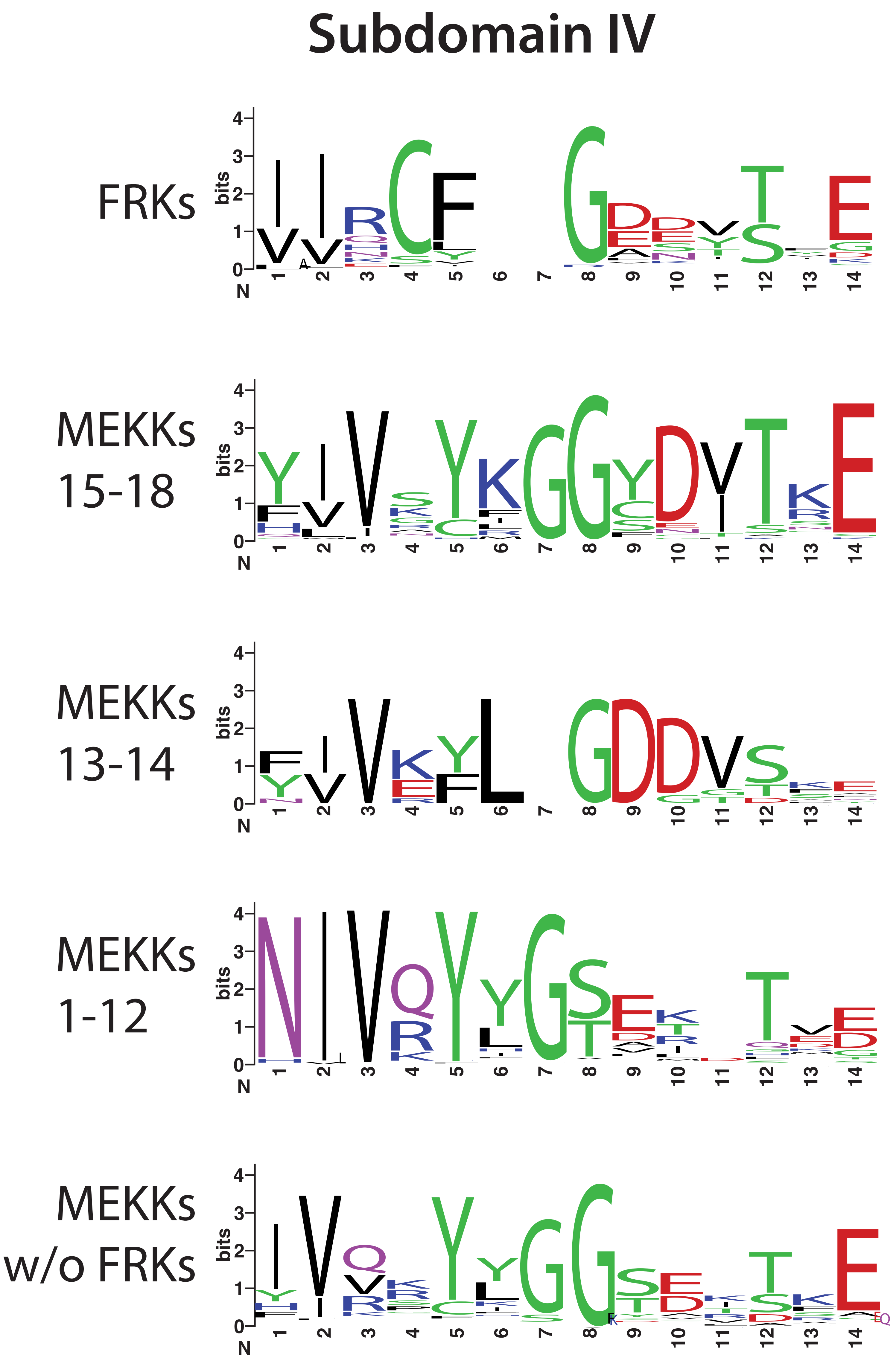
Subdomain IV
FRKs
MEKKs 15-18
MEKKs 13-14
MEKKs 1-12
MEKKs w/o FRKs
bits
N

Subdomain V

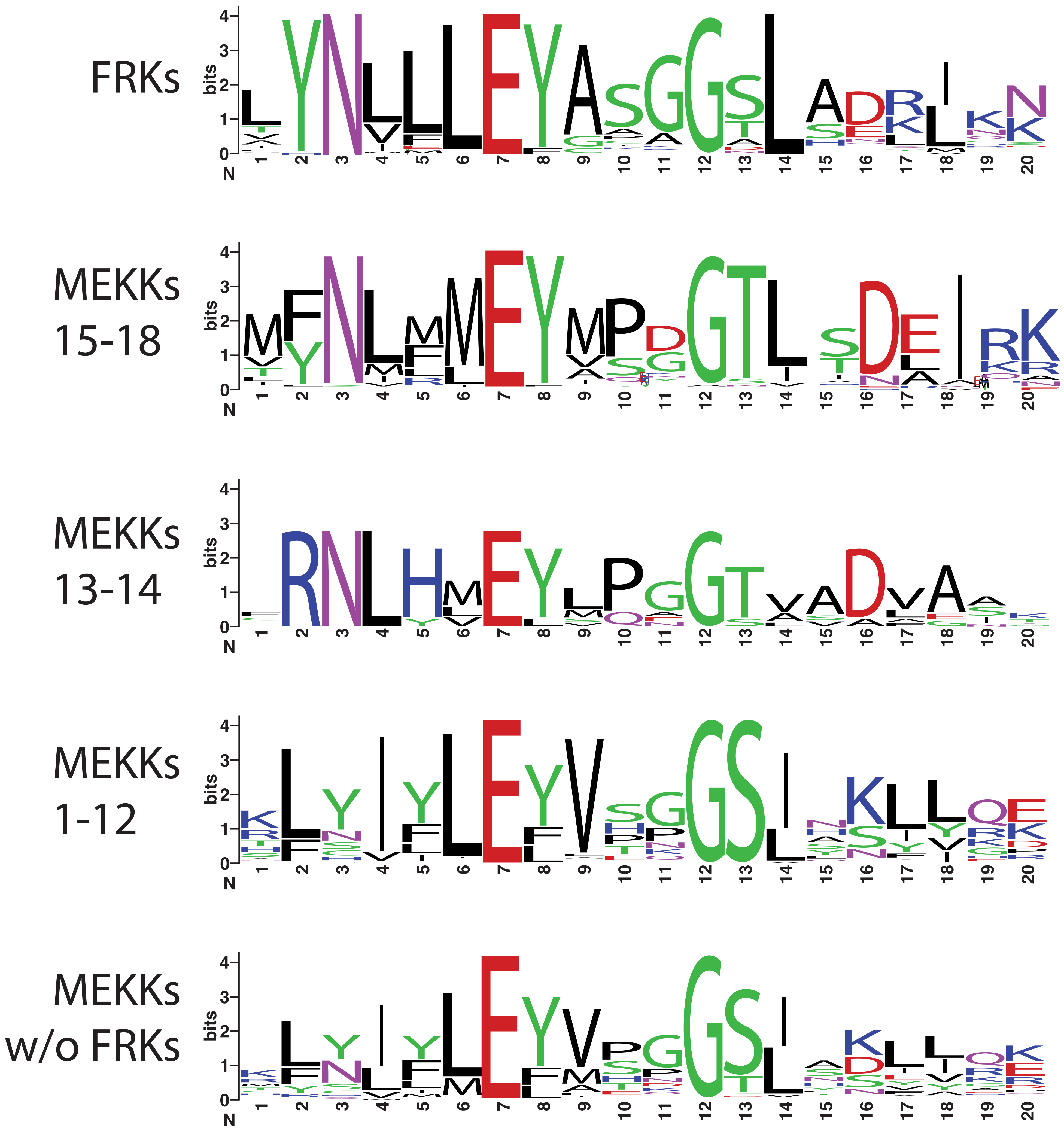

Subdomain VIa

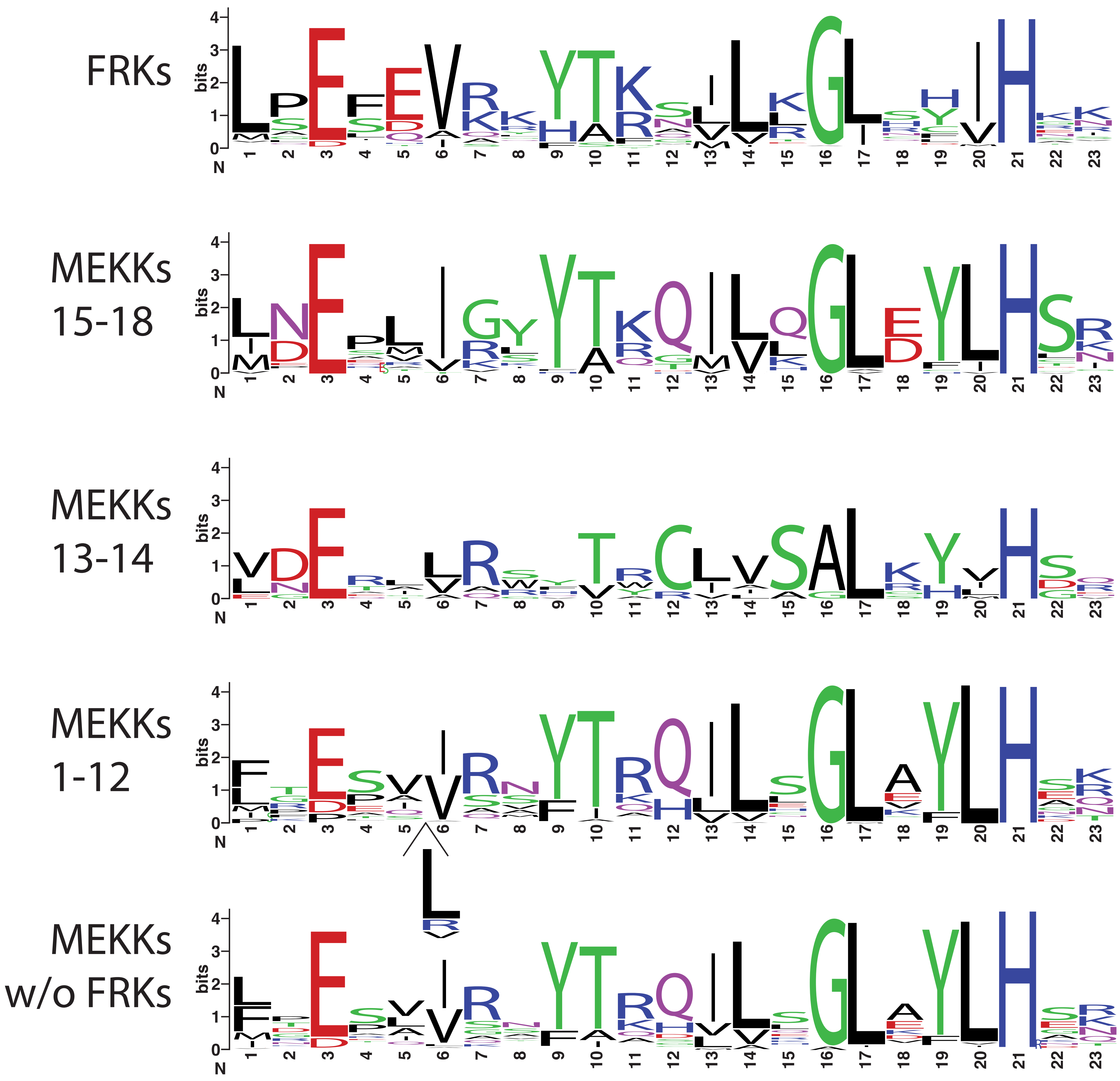

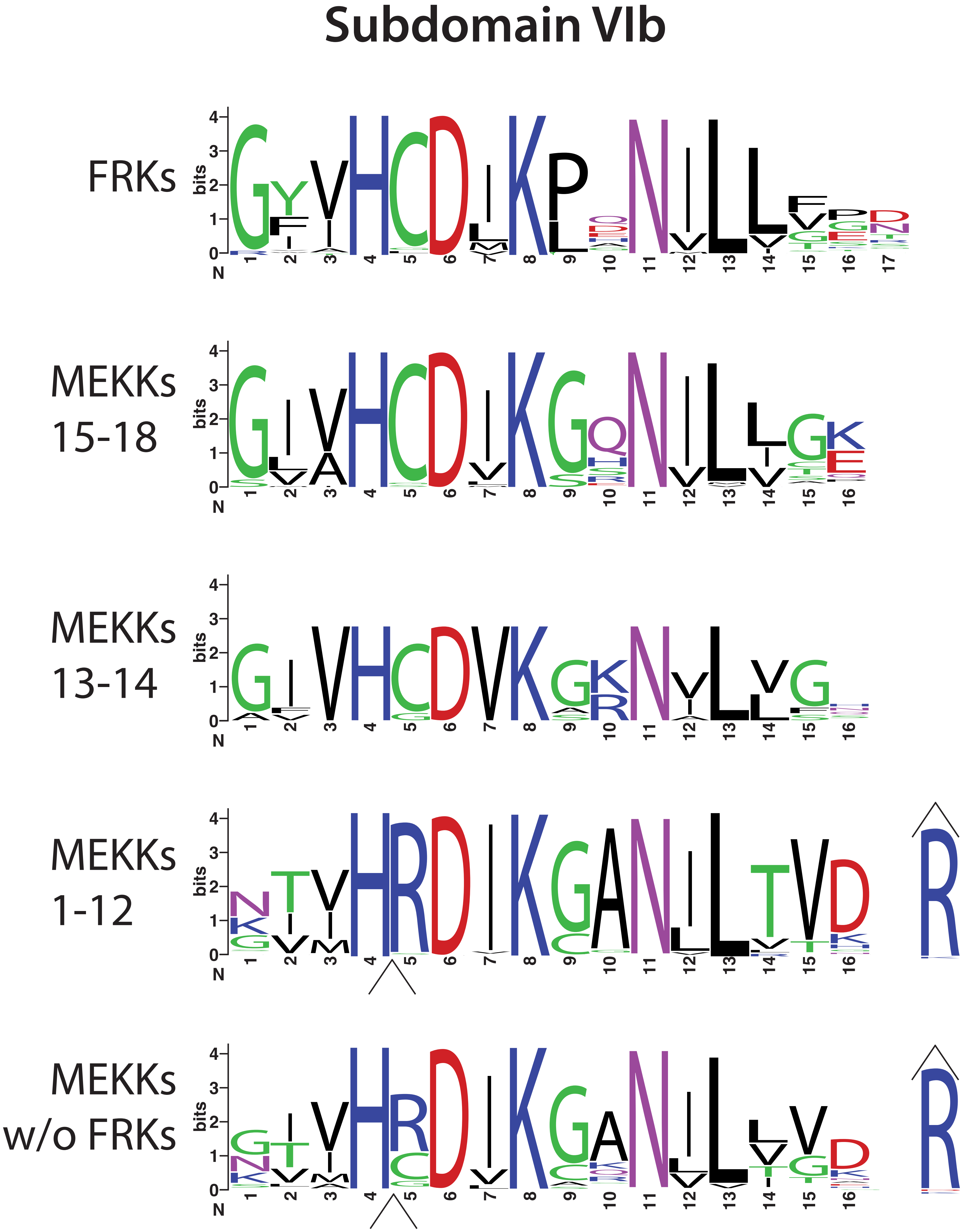

Subdomain VIb
FRKs
MEKKs 15-18
MEKKs 13-14
MEKKs 1-12
MEKKs w/o FRKs
bits
N

Subdomain VII

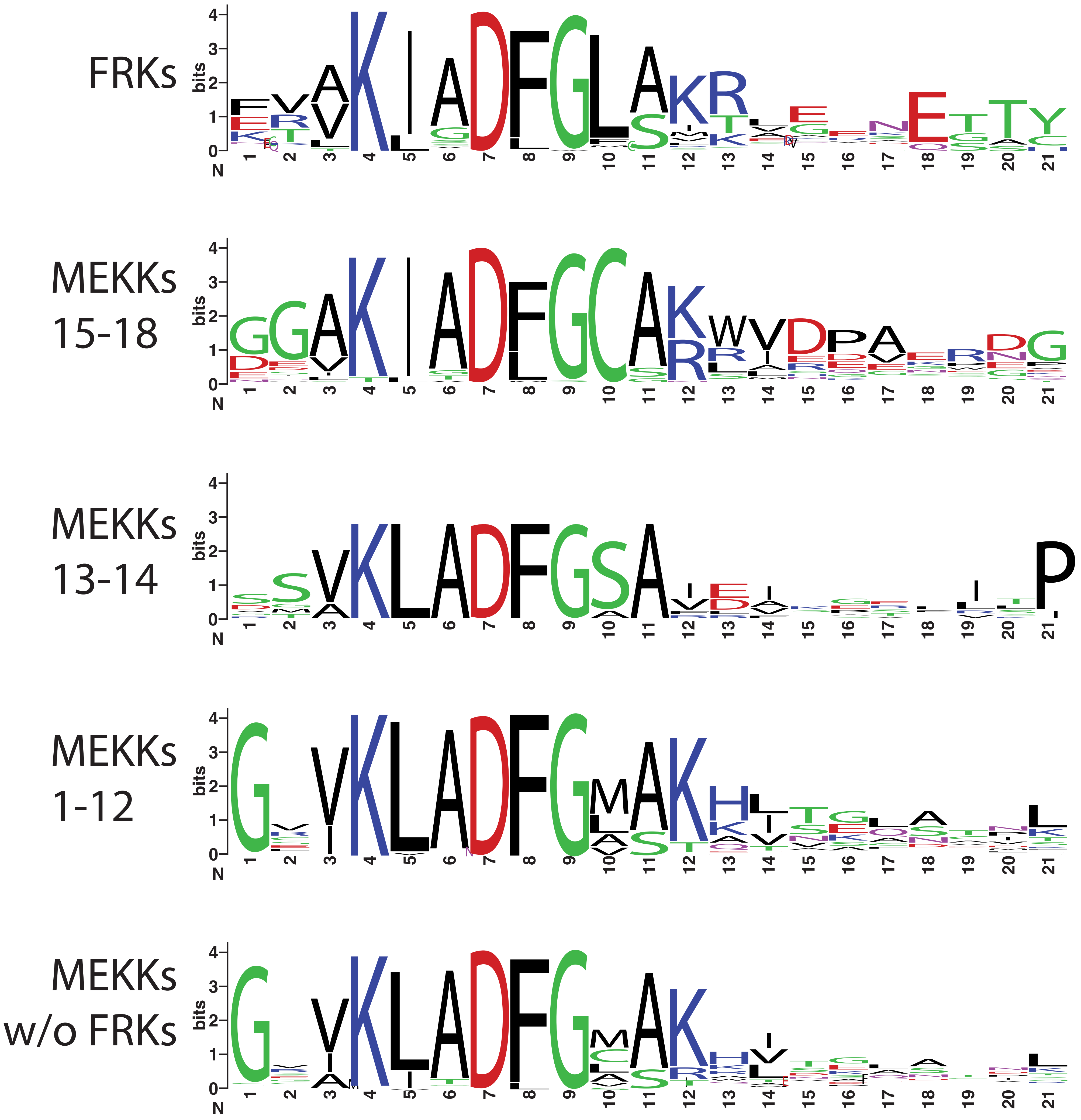

Subdomain VIII

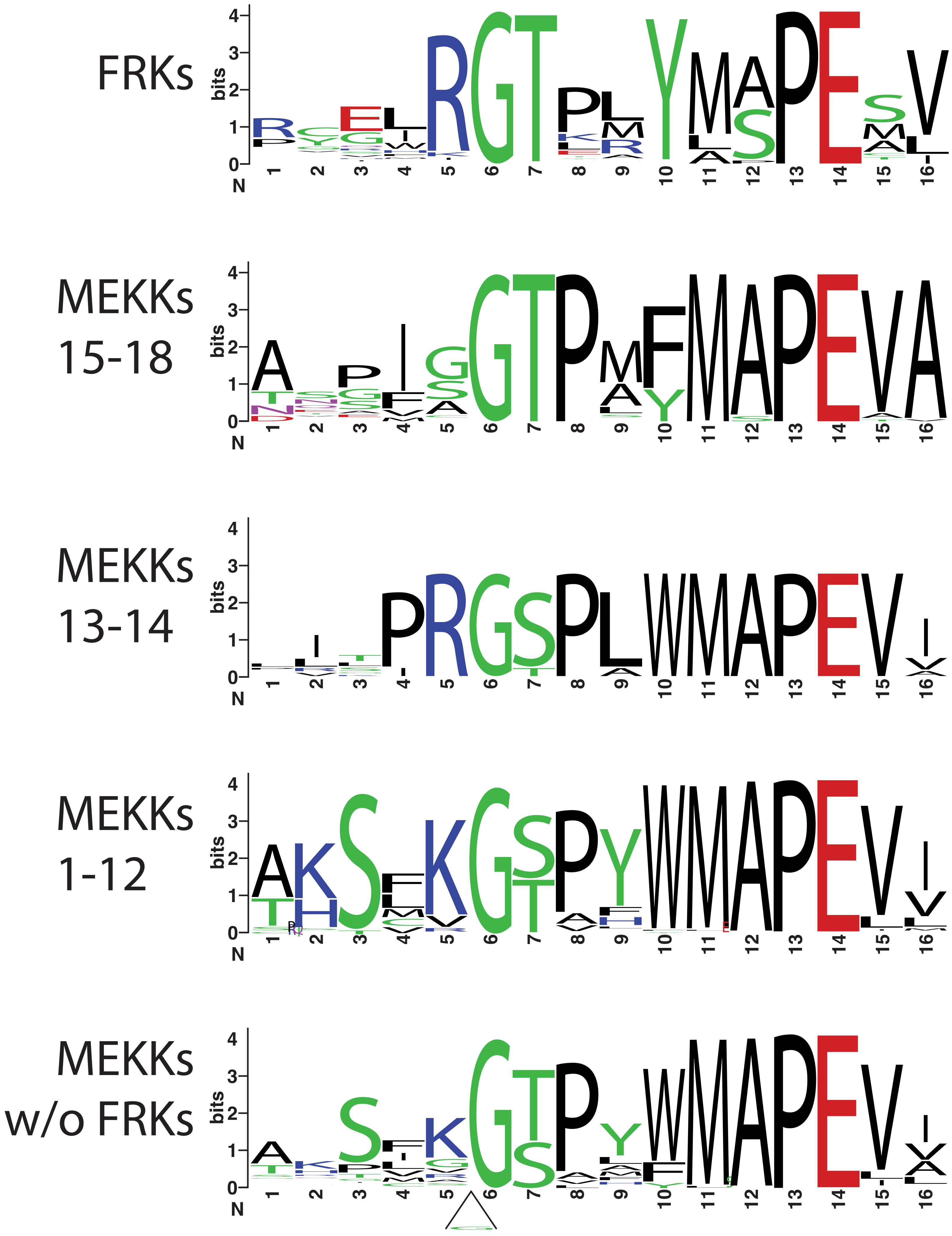

Subdomain IX

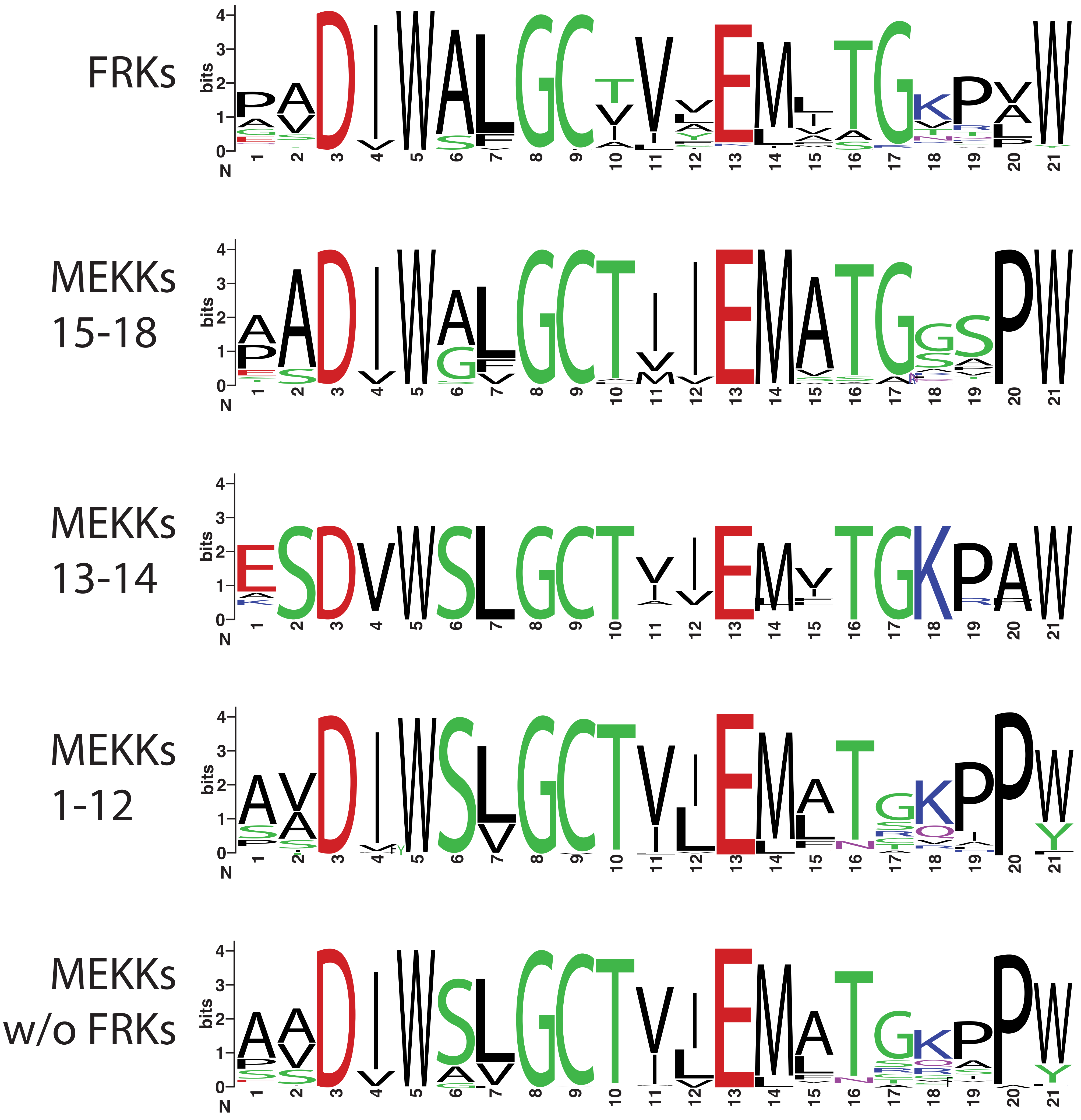

Subdomain X

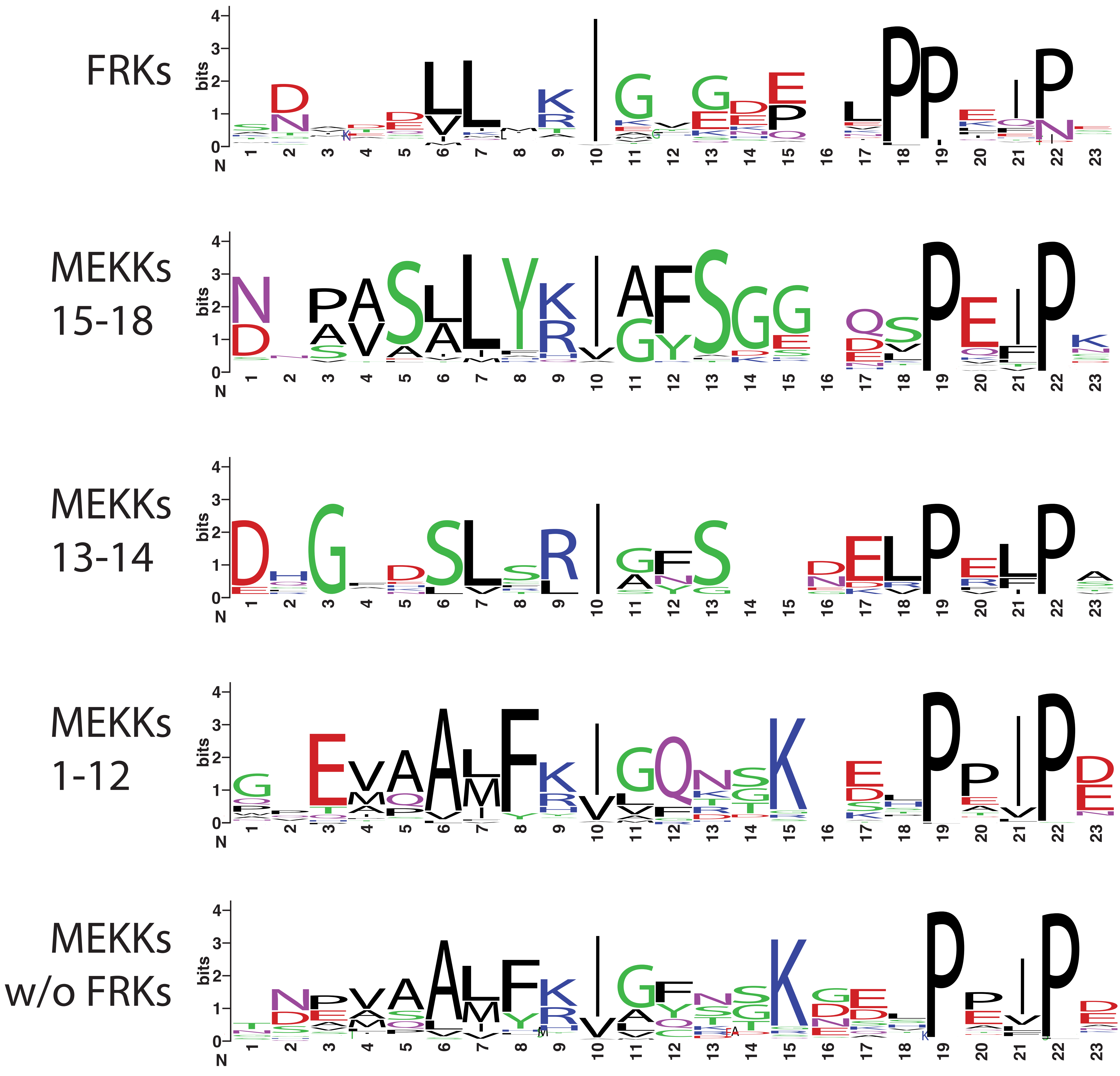

Subdomain XI

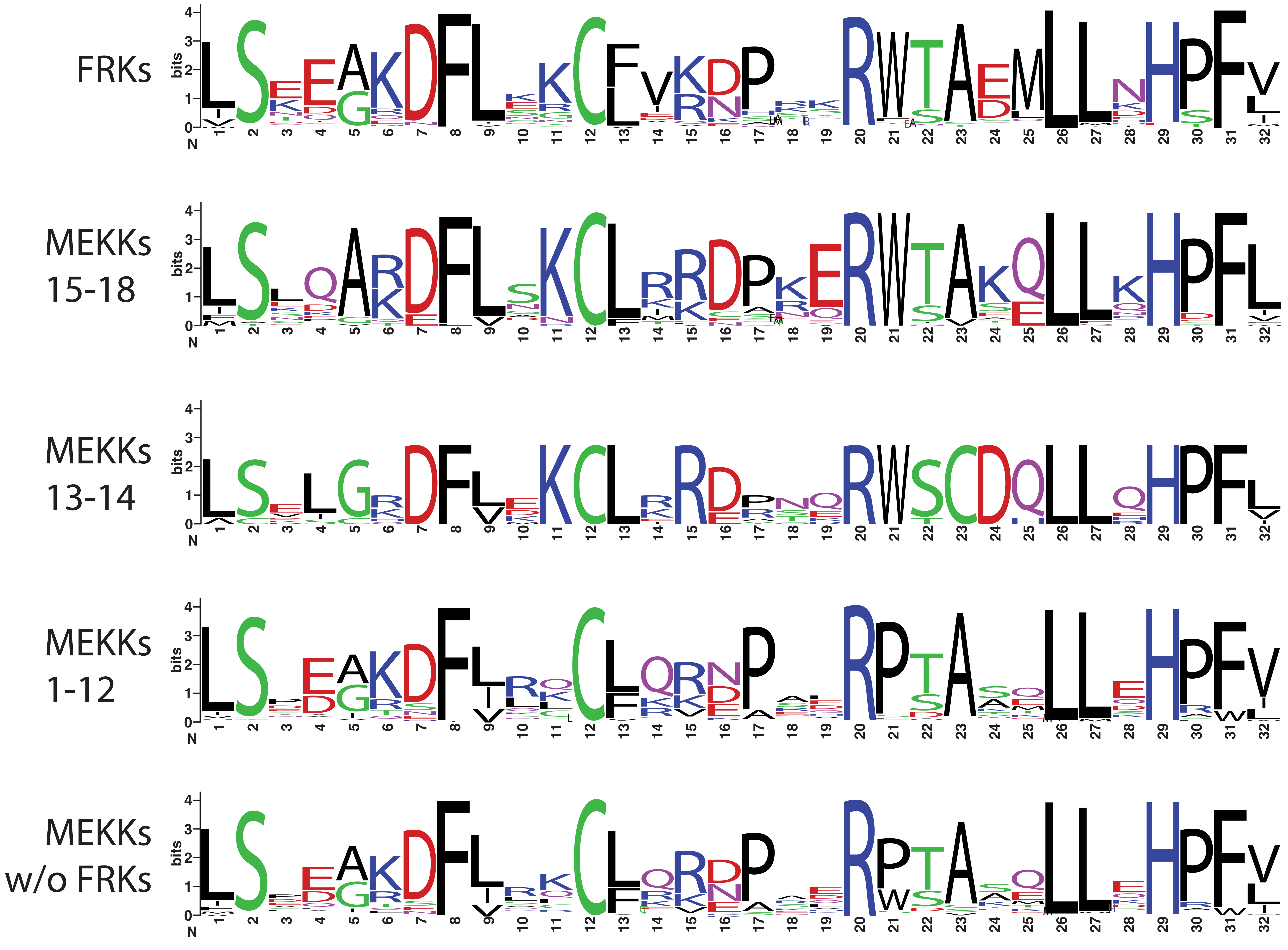

Supplement: Additional file 2: Figure S1. — Phylogenetic analysis of the MEKK subfamily in four Solanaceous species (S. chacoense, S. tuberosum, S. lycopersicum, and N. benthamiana). The kinase domain of the A. thaliana BRI1 receptor kinase was used as the outgroup to root the tree. Figure S2. Phylogenetic analysis of the whole MEKK subfamily into the 15 studied species and S. chacoense. The kinase domain of the A. thaliana BRI1 receptor kinase was used as the outgroup to root the tree. Figure S3. Phylogenetic analysis of the whole MEKK subfamily in P. abies, P. glauca and A. thaliana. Sequence from AtMPK1 was used as the outgroup to root the tree. Figure S4. Sequence logos of the 12 subdomains of the kinase catalytic domain from all FRKs orthologs, AtMAPKKK15-18 orthologs, AtMAPKKK13-14 orthologs, and AtMAPKKK1-12 orthologs. Sequence alignments of all AtMAPKKK1-12 orthologs were used to create a sequence logo using the WebLogo website (http://weblogo.berkeley.edu/logo.cgi) in order to compare the 12 kinase subdomains. The same procedure was applied to AtMAPKKK13-14 orthologs, the AtMAPKKK15-18 orthologs and all the FRK-like proteins, separately. Sequence logos were also used to compare each group (groups I to IV) of the FRK class. Figure S5. Expression analysis of members of the FRK class in four species. A. RT-PCR expression analyses of ScFRK1 to 6 from various tissues in S. chacoense. B-D. Absolute expression data taken from P. trichocarpa (B), S. lycopersicum (C), and S. tuberosum (D) available in The Bio-Analytic Resource (BAR) for plant biology, (http://bbc.botany.utoronto.ca). Columns in the heat maps are independant from each other. Lowest expression level in represented in yellow while the highest is in red. (ZIP 11579 kb) [file 12864_2015_2228_MOESM2_ESM.zip › 12864_2015_2228_add3/Additional file 2 - Figure S4.pdf]
