# Supplementary material for: Genome-wide analysis of MAPKKKs shows expansion and evolution of a new MEKK class involved in solanaceous species sexual reproduction
Source: BMC Genomics. 2015 Dec 9;16:1037. doi: 10.1186/s12864-015-2228-3 (PMC4673785; doi:10.1186/s12864-015-2228-3)
Supplement: Additional file 2: Figure S1. — Phylogenetic analysis of the MEKK subfamily in four Solanaceous species (S. chacoense, S. tuberosum, S. lycopersicum, and N. benthamiana). The kinase domain of the A. thaliana BRI1 receptor kinase was used as the outgroup to root the tree. Figure S2. Phylogenetic analysis of the whole MEKK subfamily into the 15 studied species and S. chacoense. The kinase domain of the A. thaliana BRI1 receptor kinase was used as the outgroup to root the tree. Figure S3. Phylogenetic analysis of the whole MEKK subfamily in P. abies, P. glauca and A. thaliana. Sequence from AtMPK1 was used as the outgroup to root the tree. Figure S4. Sequence logos of the 12 subdomains of the kinase catalytic domain from all FRKs orthologs, AtMAPKKK15-18 orthologs, AtMAPKKK13-14 orthologs, and AtMAPKKK1-12 orthologs. Sequence alignments of all AtMAPKKK1-12 orthologs were used to create a sequence logo using the WebLogo website (http://weblogo.berkeley.edu/logo.cgi) in order to compare the 12 kinase subdomains. The same procedure was applied to AtMAPKKK13-14 orthologs, the AtMAPKKK15-18 orthologs and all the FRK-like proteins, separately. Sequence logos were also used to compare each group (groups I to IV) of the FRK class. Figure S5. Expression analysis of members of the FRK class in four species. A. RT-PCR expression analyses of ScFRK1 to 6 from various tissues in S. chacoense. B-D. Absolute expression data taken from P. trichocarpa (B), S. lycopersicum (C), and S. tuberosum (D) available in The Bio-Analytic Resource (BAR) for plant biology, (http://bbc.botany.utoronto.ca). Columns in the heat maps are independant from each other. Lowest expression level in represented in yellow while the highest is in red. (ZIP 11579 kb) [file Additional file 2 - Figure S1.pdf › 12864_2015_2228_add3/Additional file 2 - Figure S1.pdf]

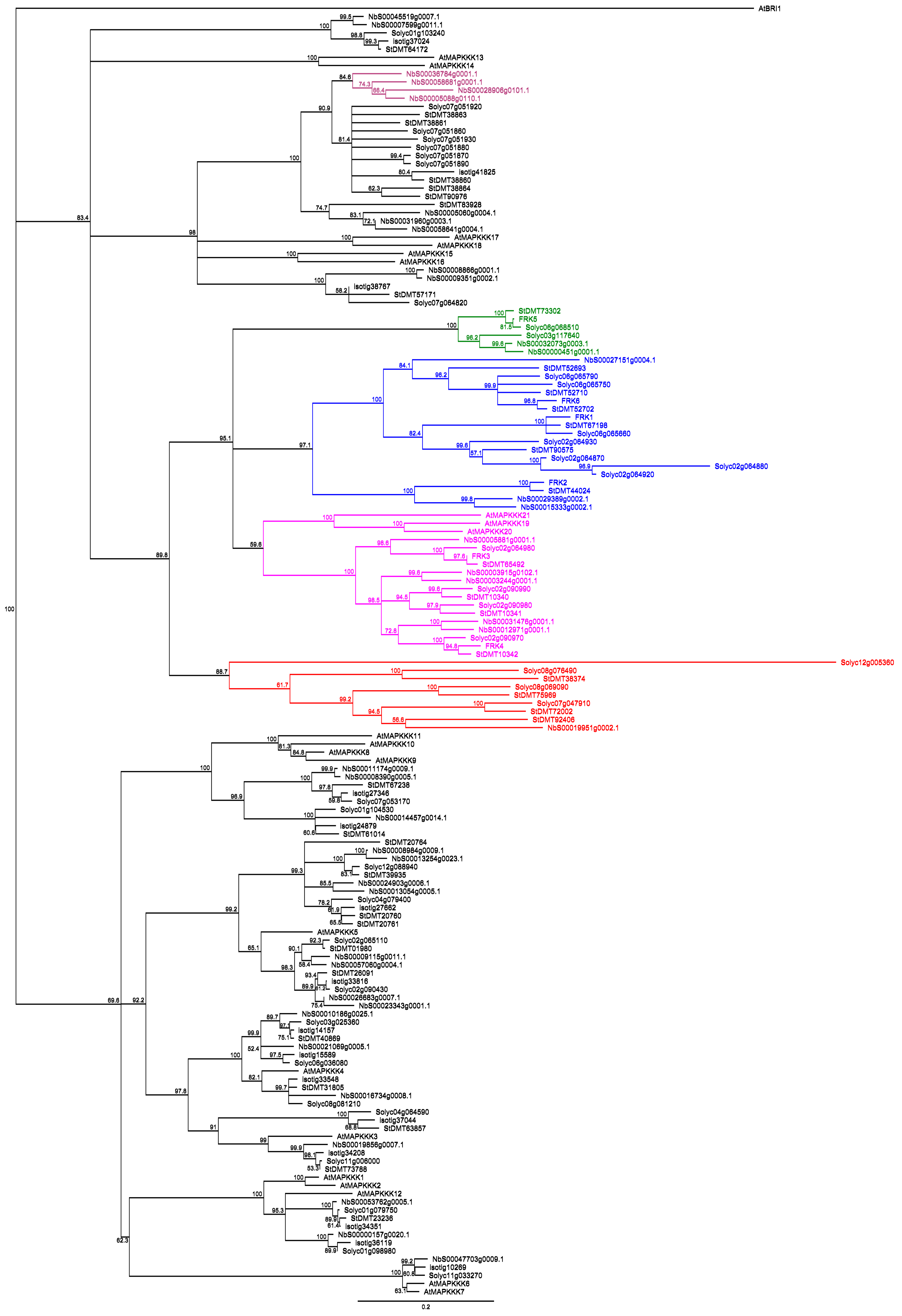

AtBRI1
NbS00045519g0007.1
NbS00007599g0011.1
Solyc01g103240
isotig37024
StDMT64172
AtMAPKKK13
AtMAPKKK14
NbS00036784g0001.1
NbS00058681g0001.1
NbS00028906g0101.1
NbS00005088g0110.1
Solyc07g051920
StDMT38863
StDMT38861
Solyc07g051860
Solyc07g051930
Solyc07g051880
Solyc07g051870
Solyc07g051890
isotig41825
StDMT38860
StDMT38864
StDMT90976
StDMT83928
NbS00005060g0004.1
NbS00031960g0003.1
NbS00058641g0004.1
AtMAPKKK17
AtMAPKKK18
AtMAPKKK15
AtMAPKKK16
NbS00008866g0001.1
NbS00009351g0002.1
isotig38767
StDMT57171
Solyc07g064820
StDMT73302
FRK5
Solyc06g068510
Solyc03g117640
NbS00032073g0003.1
NbS00000451g0001.1
NbS00027151g0004.1
StDMT52693
Solyc06g065790
Solyc06g065750
StDMT52710
FRK6
StDMT52702
FRK1
StDMT67198
Solyc06g065660
Solyc02g064930
StDMT90575
Solyc02g064870
Solyc02g064880
Solyc02g064920
FRK2
StDMT44024
NbS00029389g0002.1
NbS00015333g0002.1
AtMAPKKK21
AtMAPKKK19
AtMAPKKK20
NbS00005881g0001.1
Solyc02g064980
FRK3
StDMT65492
NbS00003915g0102.1
NbS00003244g0001.1
Solyc02g090990
StDMT10340
Solyc02g090980
StDMT10341
NbS00031476g0001.1
NbS00012971g0001.1
Solyc02g090970
FRK4
StDMT10342
Solyc12g005360
Solyc08g076490
StDMT38374
Solyc08g069090
StDMT75969
Solyc07g047910
StDMT72002
StDMT92406
NbS00019951g0002.1
AtMAPKKK11
AtMAPKKK10
AtMAPKKK8
AtMAPKKK9
NbS00011174g0009.1
NbS00008390g0005.1
StDMT67238
isotig27346
Solyc07g053170
Solyc01g104530
NbS00014457g0014.1
isotig24879
StDMT61014
StDMT20764
NbS00008984g0009.1
NbS00013254g0023.1
Solyc12g088940
StDMT39935
NbS00024903g0006.1
NbS00013054g0005.1
Solyc04g079400
isotig27662
StDMT20760
StDMT20761
AtMAPKKK5
Solyc02g065110
StDMT01980
NbS00009115g0011.1
NbS00057060g0004.1
StDMT26091
isotig33816
Solyc02g090430
NbS00026683g0007.1
NbS00023343g0001.1
NbS00010186g0025.1
Solyc03g025360
isotig14157
StDMT40869
NbS00021069g0005.1
isotig15589
Solyc06g036080
AtMAPKKK4
isotig33548
StDMT31805
NbS00016734g0008.1
Solyc08g081210
Solyc04g064590
isotig37044
StDMT63857
AtMAPKKK3
NbS00019856g0007.1
isotig34208
Solyc11g006000
StDMT73788
AtMAPKKK1
AtMAPKKK2
AtMAPKKK12
NbS00053762g0005.1
Solyc01g079750
StDMT23236
isotig34351
NbS00000157g0020.1
isotig36119
Solyc01g098980
NbS00047703g0009.1
isotig10269
Solyc11g033270
AtMAPKKK6
AtMAPKKK7
0.2
